# Elemene Injection Overcomes Paclitaxel Resistance in Breast Cancer through AR/RUNX1 Signal: Network Pharmacology and Experimental Validation

**DOI:** 10.2174/0113816128315677240620052444

**Published:** 2024-06-24

**Authors:** Xidong Gu, Leilai Xu, Yuanyuan Fu, Shuyao Fan, Tianjian Huang, Jiangting Yu, Jiaying Chen, Xinbing Sui, Xiaohong Xie

**Affiliations:** 1 Department of Breast Surgery, The First Affiliated Hospital of Zhejiang Chinese Medical University, Hangzhou 310003, Zhejiang, China;; 2 The First School of Clinical Medicine, Zhejiang Chinese Medical University, Hangzhou 310053, Zhejiang, China;; 3 Department of Medical Oncology, School of Pharmacy, The Affiliated Hospital of Hangzhou Normal University, Hangzhou 310015, Zhejiang, China

**Keywords:** Breast cancer, paclitaxel resistance, elemene injection, network pharmacology, bioinformatics, chemotherapy

## Abstract

**Background:**

Paclitaxel (PTX) is a cornerstone chemotherapy for Breast Cancer (BC), yet its impact is limited by emerging resistance. Elemene Injection (EI) has shown potential in overcoming chemotherapy resistance. However, the efficacy by which EI restores PTX sensitivity in BC and the implicated molecular mechanism remain uncharted.

**Methods:**

Network pharmacology and bioinformatic analysis were conducted to investigate the targets and mechanisms of EI in overcoming PTX resistance. A paclitaxel-resistant MCF-7 cell line (MCF-7PR) was established. The efficacy of EI and/or PTX in inhibiting cell viability was evaluated using sulforhodamine B assay, while cell proliferation was assessed using EdU staining. Furthermore, protein and gene expression analysis was performed through Western blotting and qPCR.

**Results:**

The EI containing three active components exhibited a multifaceted impact by targeting an extensive repertoire of 122 potential molecular targets. By intersecting with 761 differentially expressed genes, we successfully identified 9 genes that displayed a direct association with resistance to PTX in BC, presenting promising potential as therapeutic targets for the EI to effectively counteract PTX resistance. Enrichment analysis indicated a significant correlation between these identified targets and critical biological processes, particularly DNA damage response and cell cycle regulation. This correlation was further substantiated through meticulous analysis of single-cell datasets. Molecular docking analysis revealed robust binding affinities between the active components of the EI and the identified molecular targets. Subsequently, *in vitro* experiments unequivocally demonstrated the dose- and time-dependent inhibitory effects of the EI on both PTX-resistant and sensitive BC cell lines, effectively mitigating the resistance phenotype associated with PTX administration. Furthermore, our findings have indicated EI to effectively suppress the protein expression levels of AR and RUNX1 in MCF-7 and MCF-7PR cells under PTX treatment, as well as downregulate the mRNA expression levels of stem-like properties’ markers, KLF4 and OCT4, in these cell lines.

**Conclusion:**

Elemene Injection (EI) application has exhibited a significant capability to mitigate PTX resistance in BC, which has been achieved through targeted suppression of the AR/RUNX1 axis, revealing a key strategy to overcome chemotherapeutic resistance.

## 1 INTRODUCTION

Breast Cancer (BC) witnessed a surge in newly diagnosed cases surpassing lung cancer incidence in 2020, thereby assuming the role of the predominant form of cancer and ranking as the fifth foremost cause of cancer-related deaths worldwide [1]. Therapeutic modalities for BC encompass surgical excision, radiotherapy, and chemotherapy. Furthermore, hormone therapy, targeted therapy, and immunotherapy have significantly impacted the management of BC, offering more personalized and potentially less invasive options for patients [2]. These treatments are tailored to address the heterogeneity of BC, considering the distinct molecular subtypes and individual patient characteristics. Hormone therapy, particularly for hormone receptor-positive BC, has seen advancements with the development of selective estrogen receptor modulators and aromatase inhibitors, which have improved patient outcomes [3]. Targeted therapies, such as HER2-directed agents, have transformed the treatment of HER2-positive BC, leading to significant improvements in survival rates [4]. Moreover, the advent of immunotherapy has introduced a new frontier in BC treatment, harnessing the patient's immune system to combat cancer cells, with notable success in triple-negative BC [5-8].

Chemotherapy stands as a cornerstone treatment strategy, validated for its efficacy in restraining tumor proliferation and extending patient survival. Taxanes, exemplified by Paclitaxel (PTX), have garnered regulatory approval for employment as chemotherapeutic agents in the management of early-stage and advanced metastatic BC [9]. Nevertheless, the effectiveness of taxane-derived regimens in addressing metastatic BC varies between 30% and 70% [10]. Literature reports that more than 90% of non-responsive patients exhibit either intrinsic or acquired resistance towards this therapeutic modality, attributable to genetic alterations or adaptive mechanisms [11]. Hence, the identification and comprehension of putative mechanisms governing chemotherapy resistance assume paramount significance, holding the potential to enhance BC treatment outcomes.

Western medicine provides valuable approaches for the diagnosis and treatment of tumors, but it can also result in severe adverse reactions. Traditional Chinese Medicine (TCM) holds promise in enhancing the quality of life of patients. However, the advancement of TCM and its integration with Western medicine, which has the potential to become a mainstream therapeutic approach for cancer patients [12, 13], is hindered by the absence of an effective evaluation system. Elemene, a lipophilic plant medicine derived from *Curcuma wenyujin*, has garnered significant attention as an anticancer agent [14]. The active ingredients in Elemene Injection (EI) encompass β-elemene, γ-elemene, and σ-elemene, with accompanying excipients, such as cellulose, cholesterol, alcohol, disodium hydrogen phosphate, and dihydrogen phosphate [15]. Among these components, β-elemene serves as the primary active compound. Elemene has demonstrated broad-spectrum anticancer effects in various malignancies, including BC, gastric cancer, and ovarian cancer, making it a noteworthy contribution to the field of biomedicine [16]. Notably, elemene exhibits lower toxicity to normal cells compared to conventional chemotherapeutic drugs, thereby being considered a safe option [16]. Clinically, β-elemene has been utilized for radiosensitization and chemotherapy in different types of tumors, effectively reversing drug resistance [17, 18]. However, the potential of EI to reverse PTX resistance in BC remains uncertain.

In this study, by integrating network pharmacology approaches with rigorous experimental validation, we have, for the first time, elucidated the mechanism by which EI effectively reverses PTX resistance in BC, specifically through targeted inhibition of the Androgen Receptor (AR)/RUNX1 signaling axis. This finding introduces a novel strategy for overcoming chemoresistance and underscores the potential clinical utility of EI in combating PTX resistance. Moreover, our results not only affirm the therapeutic promise of EI in this context, but also address a significant knowledge gap concerning the role of the AR/RUNX1 pathway in regulating BC chemoresistance.

## 2 MATERIALS AND METHODS

### 2.1 Differential Expression Analysis

The differential expression analysis was conducted utilizing the GEO2R tool (https://www.ncbi.nlm.nih.gov/geo/geo2r/), which is a widely employed method for such purposes, on the GSE144113 dataset. This dataset encompasses transcriptomic sequencing data of HS578T and BAS BC cells, along with their respective PTX-resistant cell lines. The identification of differentially expressed genes was achieved by applying a stringent statistical threshold of an adjusted *p*-value less than 0.05.

### 2.2 Network Pharmacology Analysis

The target identification and prediction of EI were conducted utilizing the Traditional Chinese Medicine Systems Pharmacology Database and Analysis Platform (TCMSP, https://old.tcmsp-e.com/tcmsp.php) [19], Comparative Toxicogenomics Database (CTD, https://ctdbase.org/) [20], and SwissTargetPrediction (STP, http://www.swisstargetprediction.ch/) [21] databases or tools. The protein-protein interaction network was extracted from the STRING database (https://cn.string-db.org/). Subsequently, enrichment analysis was performed using the ClueGO plugin within the Cytoscape software [22], encompassing the assessment of biological processes, cellular components, molecular functions, KEGG pathways, WikiPathways, and Reactome pathways.

### 2.3 Single-cell Sequencing Analysis

The cancerSEA resource (http://biocc.hrbmu.edu.cn/Cancer SEA/) offers an extensive compilation of functional state maps specifically designed for the analysis of single-cell data in cancer research [23]. It enables the investigation and identification of the functional states associated with different cancer types within the target gene list. In this study, a comprehensive set of potential target genes was imported and evaluated to determine their correlations with diverse cancer functional states. Furthermore, a meticulous examination of their functional states was conducted on five distinct single-cell datasets pertaining to BC (EXP0052-EXP0055).

### 2.4 Molecular Docking Analysis

Structural coordinates for the target proteins, specifically AR (PDB ID: 1T5Z), Cyclin-dependent Kinase inhibitor 1B (CDKN1B; PDB ID: 5UQ3), Interleukin-6 (IL6; PDB ID: 4J4L), Carboxylesterase 1 (CES1; PDB ID: 2HRQ), Peroxisome Proliferator-activated Receptor Delta (PPARD; PDB ID: 2GWX), Cyclin-dependent Kinase 1 (CDK1; PDB ID: 4Y72), Cannabinoid Receptor 1 (CNR1; PDB ID: 5XR8), and Tumor Protein p53 (TP53; PDB ID: 1MA3), were retrieved from the Protein Data Bank (PDB) database (https://www.rcsb.org/). Conversely, the chemical structures of the investigational compounds were sourced from the PubChem database (https://pubchem.ncbi.nlm.nih.gov/), facilitating subsequent molecular docking analyses. The PyMOL software was employed to eliminate organic solvents, water molecules, and non-target protein components from the protein structures. Subsequently, the structures were subjected to hydrogenation and charge calculation before being saved for docking analysis. The getBox plugin was utilized to generate molecular docking grids, and the AutoDock Vina software was employed to execute molecular docking simulations based on the Lamarckian genetic algorithm. Ultimately, the outcomes of the docking procedures were visually inspected and analyzed through PyMOL visualization tools.

### 2.5 Cell Culture and Reagents

The MCF-7 cell lines employed in the study were obtained from EK-Bioscience (Shanghai, China). The breast adenocarcinoma cells (MCF-7) were cultured in RPMI 1600 medium (Invitrogen, Carlsbad, CA, USA) and supplemented with 10% Fetal Bovine Serum (FBS), penicillin, and streptomycin. Incubation of the cells was carried out in a controlled environment with 5% CO_2_ at 37°C. Primary antibodies specifically targeting AR, RUNX1, and GAPDH were obtained from Abcam Shanghai Trading Co., Ltd (Abcam, Shanghai, China). AR agonist Dihydrotestosterone (DHT) was purchased from Sigma-Aldrich and diluted in ethanol, PTX (Cell Signaling Technology, Danvers, MA, USA; #98075), and Dimethyl Sulfoxide (DMSO). EI was obtained from Dalian Holley Kingkong Pharmaceutical (Dalian, China).

To establish a PTX-resistant subline, MCF-7 cells were exposed to gradually increasing concentrations of PTX for a duration exceeding 6 months, resulting in the emergence of a PTX-resistant subline known as MCF-7PR. Sulforhodamine B (SRB) assay was used to evaluate the cytotoxicity of PTX in these cell lines and to confirm the resistance at different time points during the process.

### 2.6 Cytotoxicity Studies

SRB assay was adopted for evaluating the cytotoxicity and cell viability. Briefly, the cells were seeded into a 96-well plate at a density ranging from approximately 2000 to 4000 cells per well. After incubating overnight, the cells were treated with the respective reagents (EI and/or PTX). The treatment duration was set at 24, 48, and 72 hours for EI, and 72 hours for PTX. Following treatment, the cells were fixed with 200 μl of 10% trichloroacetic acid. Subsequently, they underwent washing and staining steps using a 0.4% SRB dye solution supplemented with 0.1% acetic acid. After incubating at room temperature for 1 hour, the cells were washed three times with 1% acetic acid and air-dried. To dissolve the dye bound to proteins, a tris alkaline solution (10 mM) was added to each well, and the resulting optical density was measured using a plate reader (BioTek Instruments, VT) following established protocols [24].

### 2.7 EdU Labeling and Staining

BC cells grown on glass coverslips were treated with immunostaining using a concentration of 10 μmol/L EdU (Click-iT EdU Alexa Fluor), according to the instructions provided by the manufacturer (Thermo Fisher Scientific #C10337). After that, the cells were washed multiple times with a solution of PBS containing 0.5% Triton X-100 and fixed using Prolong Gold and DAPI (4',6- diamidino-2-phenylindole). The fluorescence emitted by the labeled cells was observed using a Leica DM5000 B fluorescence microscope. Image analysis was performed using ImageJ software.

### 2.8 Quantitative RT-PCR

RT-PCR was employed for gene expression analysis. Briefly, RNA extraction was executed using TRI reagent (MRC, Cincinnati, USA), and 1 μg of the isolated total RNA was utilized for cDNA synthesis *via* M-MLV reverse transcriptase (Promega). Subsequently, a SYBR Green-based quantitative gene expression analysis was carried out with Taq polymerase from Invitrogen (Thermo Fisher Scientific) on an Applied Biosystems StepOne platform (Thermo Fisher Scientific). The relative gene expression levels were determined by applying the 2^–∆∆Ct^ method, with normalization to GAPDH expression. Primer sequences were as follows: KLF4, CGAACCCACACAGGTGAGAA (F), TACGGTAGTGCCTGGTCAGTTC (R), OCT4, CAGGCCCGAAAGAGAAAGC (F), CCACACTGGACCACATGGT (R), GAPDH, GCACCACCAACTGCTTAGCA (F), GTCTTCTGGGTGGCAGTGATG (R).

### 2.9 Western Blot Assay

Western blot analysis was conducted to quantify the expression of the target protein in cellular extracts. Briefly, following lysis of cells in RIPA buffer complemented with protease inhibitors, protein concentrations were determined using the Bio-Rad protein assay kit (Bio-Rad Laboratories, Hercules, CA, USA). Equal quantities (30μg) of protein samples, alongside a protein ladder, were loaded onto a prepared polyacrylamide gel for SDS-PAGE electrophoresis, and resolved proteins were transferred to the PVDF membrane. The membrane was then incubated overnight at 4^o^C with a primary antibody against AR, RUNX1, and GAPDH, washed thoroughly with TBST, and subsequently exposed to a species-appropriate HRP-conjugated secondary antibody for 1-2 hours at room temperature. Protein bands were visualized using an Enhanced Chemiluminescence (ECL) substrate and captured *via* X-ray film or a chemiluminescence imaging system. Band intensities were quantified using ImageJ, with normalization to the internal control protein (GAPDH) to correct for loading inconsistencies, ensuring an accurate comparison of protein expression levels across different samples.

### 2.10 Statistical Analysis

The statistical analysis was conducted using GraphPad Prism v9.0 software. The data are expressed as mean ± Standard Deviation (SD) derived from three distinct experimental trials. A two-sample t-test was employed to compare the two groups. All experiments were performed at least three times before analyzing the statistical significance. A *p*-value of less than 0.05 was considered statistically significant.

## 3 RESULTS

### 3.1 Identification of Genes Associated with PTX Resistance in BC

**Fig. (1):**
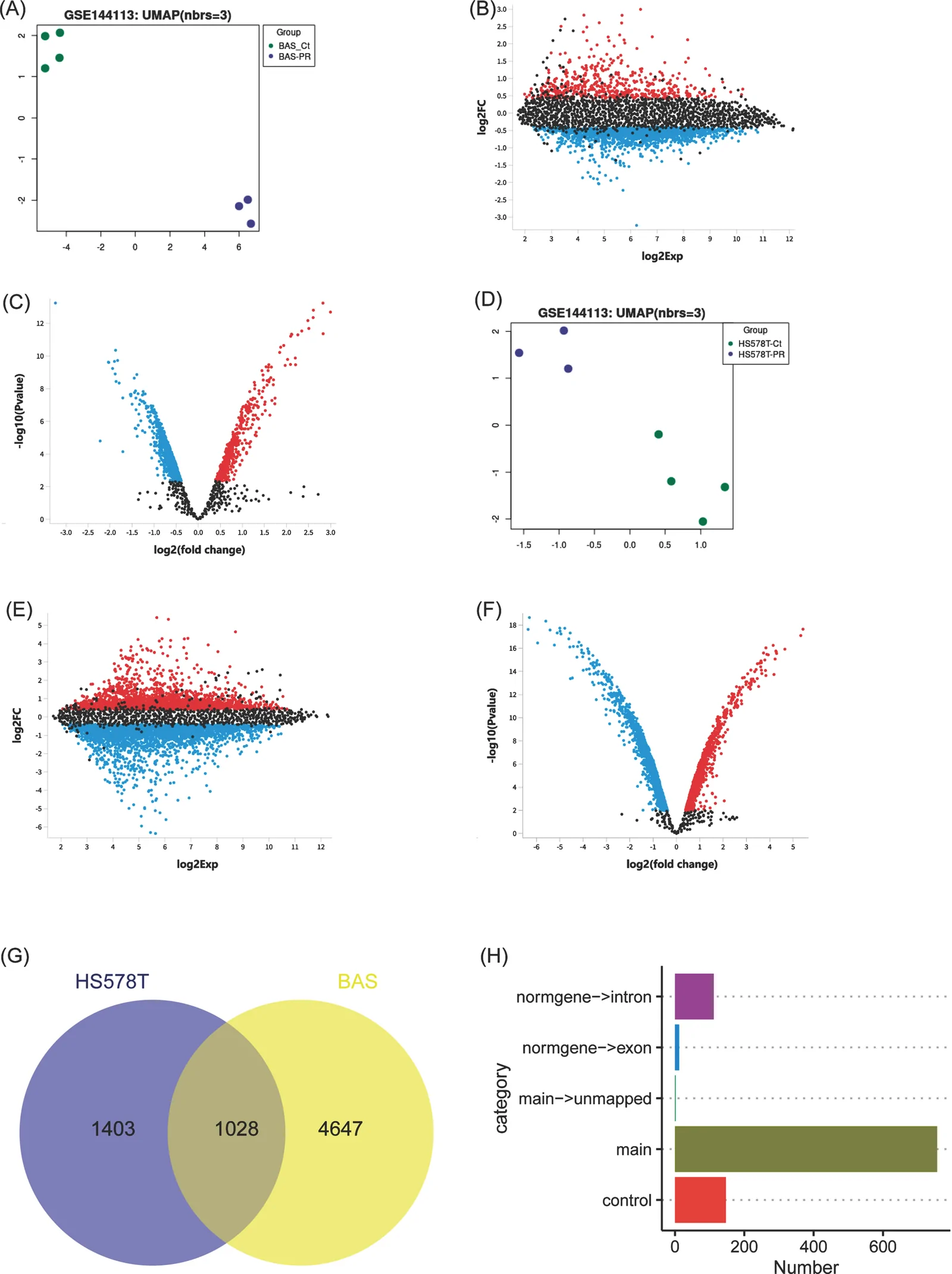
Identification of PTX-resistant related genes in breast cancer based on GSE144113. (**A**-**C**) Analysis of breast cancer BAS cell lines based on GEO2R. (**D**-**F**) Analysis of breast cancer HS578T cell lines based on GEO2R. (**G**) Venn diagram of differentially expressed genes in BAS and HS578T cell lines. (**H**) Annotation of differentially expressed probes.

In order to elucidate the genetic factors contributing to PTX resistance in BC, we conducted differential expression analysis on the GEO dataset GSE144113. This dataset encompasses transcriptome sequencing data of HS578T and BAS BC cell lines, along with their respective PTX-resistant cell lines. Through rigorous differential analysis, we identified 2431 probes exhibiting differential expression between the HS578T and HS578T-PR cell lines (Fig. **1A**-**C**), as well as 5675 probes displaying differential expression between the BAS and BAS-PR cell lines (Fig. **1D**-**F**). Subsequently, by integrating these probes sets, we consolidated a total of 1028 differentially expressed probes (Fig. **1G**). Following comprehensive annotation, we identified a subset of 762 genes exhibiting significant differential expression (Fig. **1H**, Table **S1**).

### 3.2 Potential Targets and Mechanisms of EI in Reversing PTX Resistance in BC

**Fig. (2):**
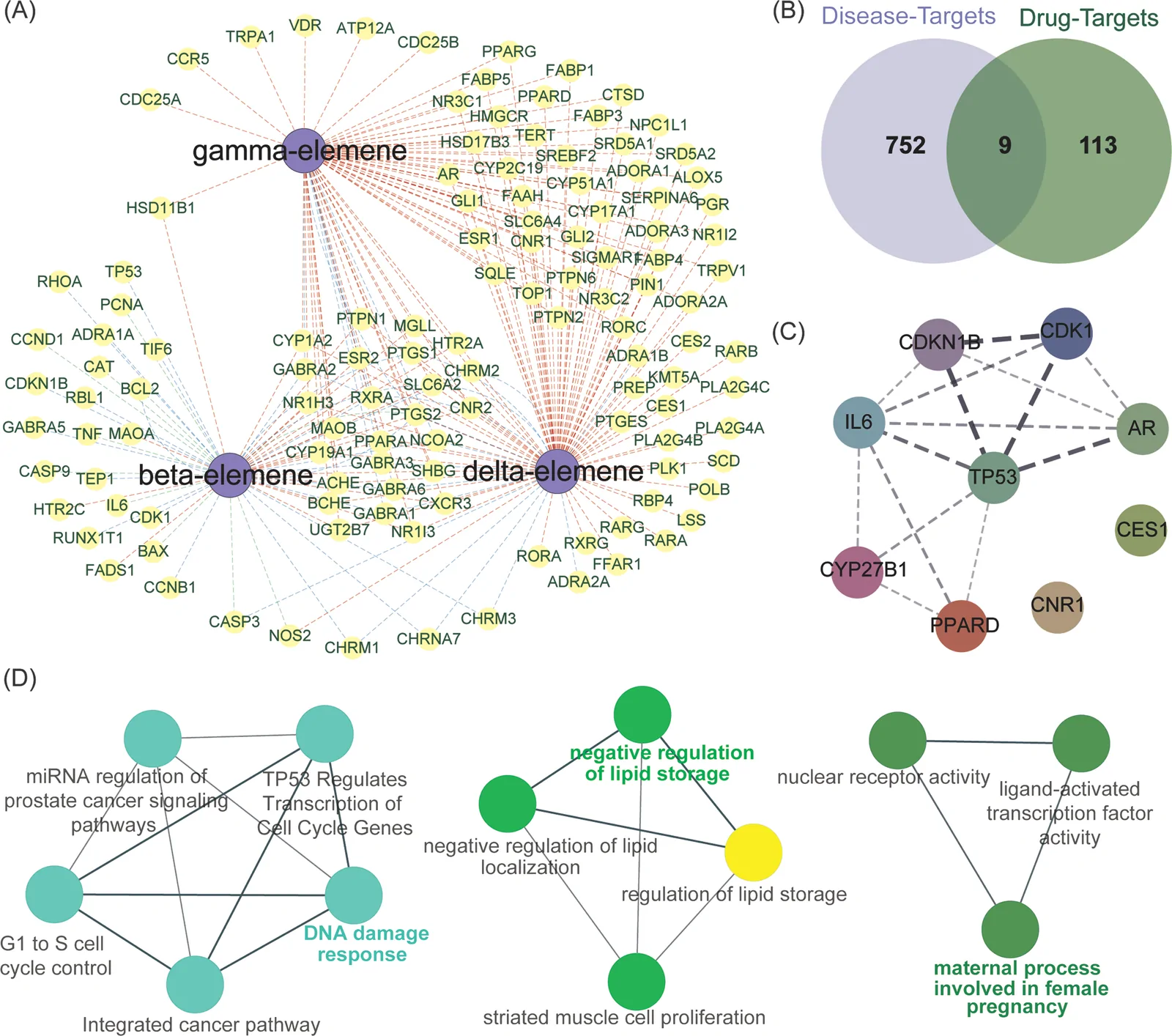
Potential targets and mechanisms of EI in reversing PTX resistance in breast cancer. (**A**) Network diagram illustrating the active ingredients and targets of EI. (**B**) Venn diagram depicting the overlap between genes associated with PTX resistance in breast cancer and EI targets. (**C**) Potential targets of EI in reversing PTX resistance in breast cancer. (**D**) Enrichment analysis of candidate targets based on ClueGO.

Fig. (**2A**) depicts the comprehensive network diagram elucidating the intricate relationships between the bioactive constituents of EI and their corresponding targets. The diagram encompasses three distinct molecular configurations of EI, alongside a comprehensive repertoire of 122 potential targets (Table **S2**). By intersecting these targets with genes specifically associated with PTX resistance in BC, a refined selection of nine potential targets emerged (Fig. **2B**), thereby revealing 15 pertinent protein-protein interaction profiles (Fig. **2C**). Subsequent enrichment analysis unveiled the involvement of these targets in pivotal pathways encompassing DNA damage response, cell cycle regulation, and lipid storage regulation, further highlighting their noteworthy attributes, such as nuclear receptor activity and transcription factor functionality (Fig. **2D**).

### 3.3 Functional Analysis of Potential Targets based on Single-cell Sequencing Data

**Fig. (3):**
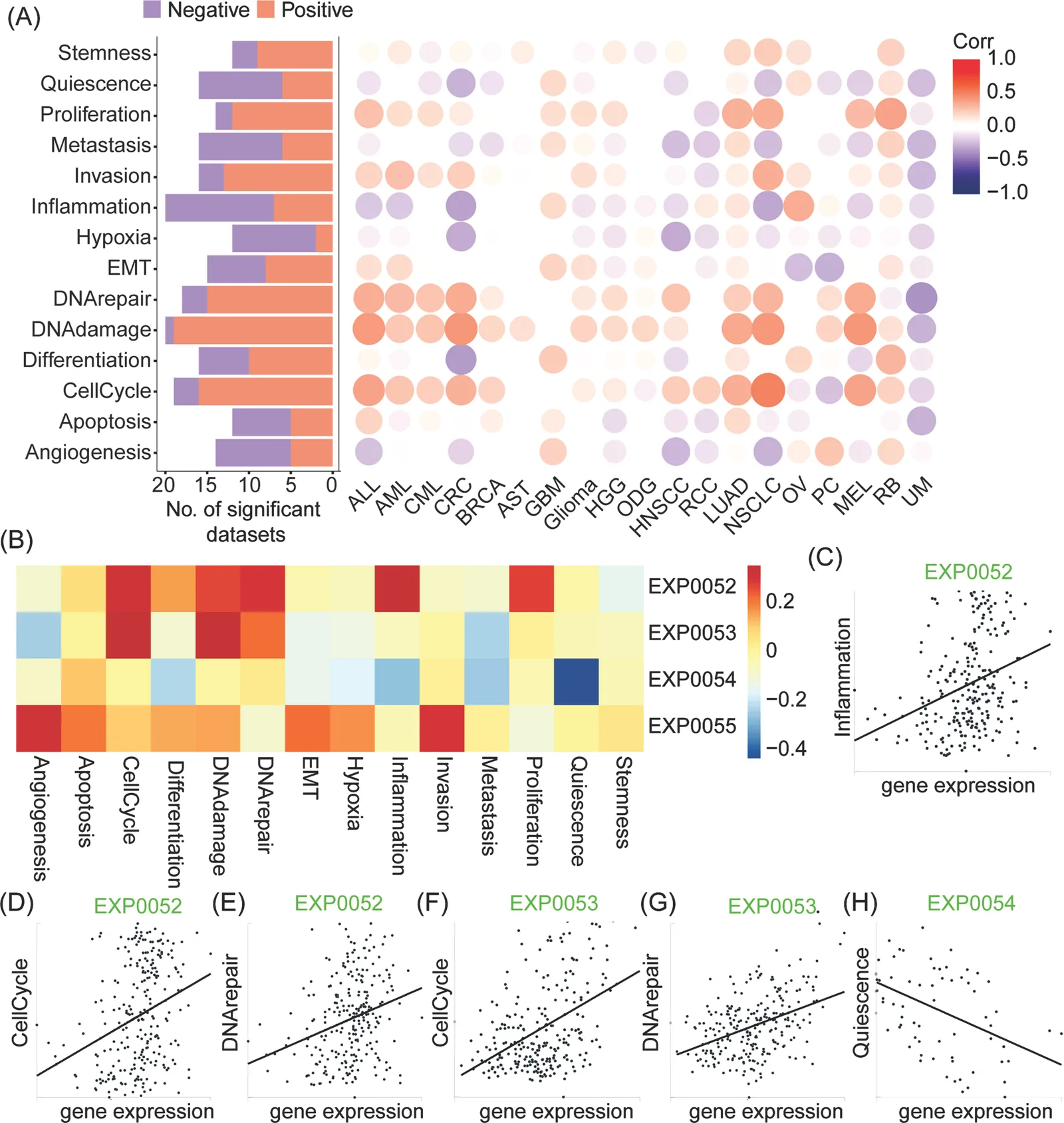
Functional analysis of potential target genes based on the CancerSEA database. (**A**) Functional correlation analysis of potential target gene sets across 19 different types of cancers. (**B**) Functional correlation analysis of potential target gene sets in 5 single-cell datasets of breast cancer. (**C**-**H**) Scatter plots showing the correlation between expression of potential target gene sets and breast cancer-related functions.

To further evaluate the functional significance of these potential targets in cancer, we employed the CancerSEA web tool. The findings demonstrated that across 19 distinct cancer types, a substantial number of datasets exhibited a statistically significant positive correlation between the potential targets and processes encompassing DNA repair, DNA damage, cell cycle progression, and cellular proliferation (Fig. **3A**). Notably, in BC, the expression patterns of these potential targets exhibited a significant positive association with inflammatory responses, cell cycle dynamics, and DNA repair mechanisms, while concurrently displaying a significant negative correlation with cellular quiescence (Fig. **3B**-**H**).

### 3.4 Validation of Molecular Docking Interactions between Active Compounds and Target Molecules

**Fig. (4):**
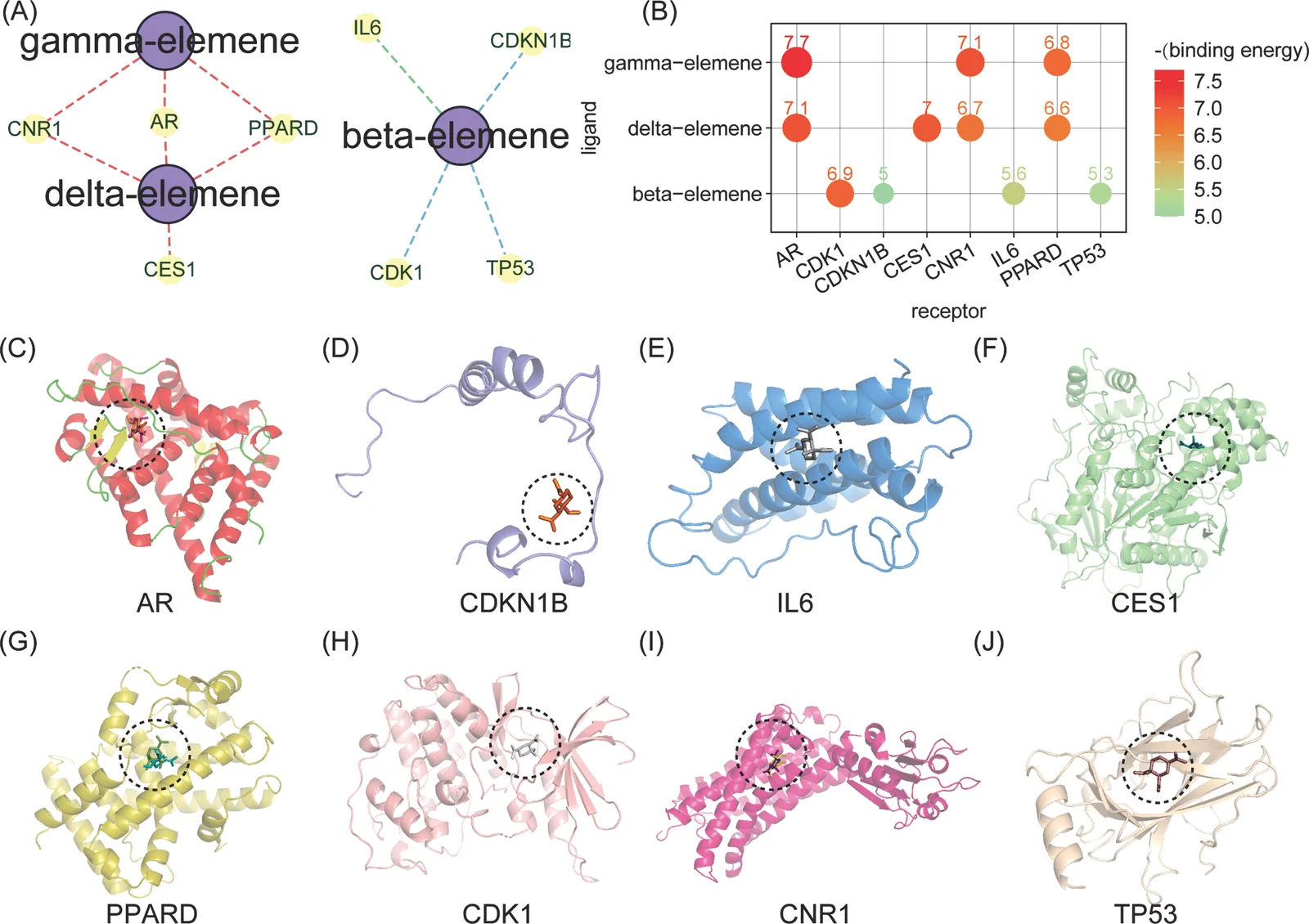
Molecular docking assessment of the binding affinity between active ingredients and their respective targets. (**A**) Network relationship between ingredients and potential targets. (**B**) Docking affinity energies of ingredients with potential targets. (**C**-**J**) 3D docking results depicting the interaction between ingredients and potential targets.

To assess the affinity between active ingredients and target molecules, we extracted the potential target-ingredient network from the ingredient-target network of the EI (Fig. **4A**). Subsequently, a comprehensive molecular docking analysis was conducted on the paired ingredient-target combinations. Our findings revealed all docking binding energies to be ≤ -5 kcal/mol (Fig. **4B**). Notably, the gamma-elemene and AR pairing exhibited the lowest binding energy, measuring at 7.7 kcal/mol. This observation implies a significant potential for strong binding interactions between the active ingredients and their respective targets. Furthermore, Fig. (**4C**-**J**) visually illustrate the docking structures of the active ingredients with the target molecules. Specifically, the AR, CNR1, and PPARD molecules were found to bind to two distinct configurations of elemene, with neighboring binding domains marked by distinguishing colors.

### 3.5 Reversal of PTX Resistance in BC through the Administration of EI

**Fig. (5):**
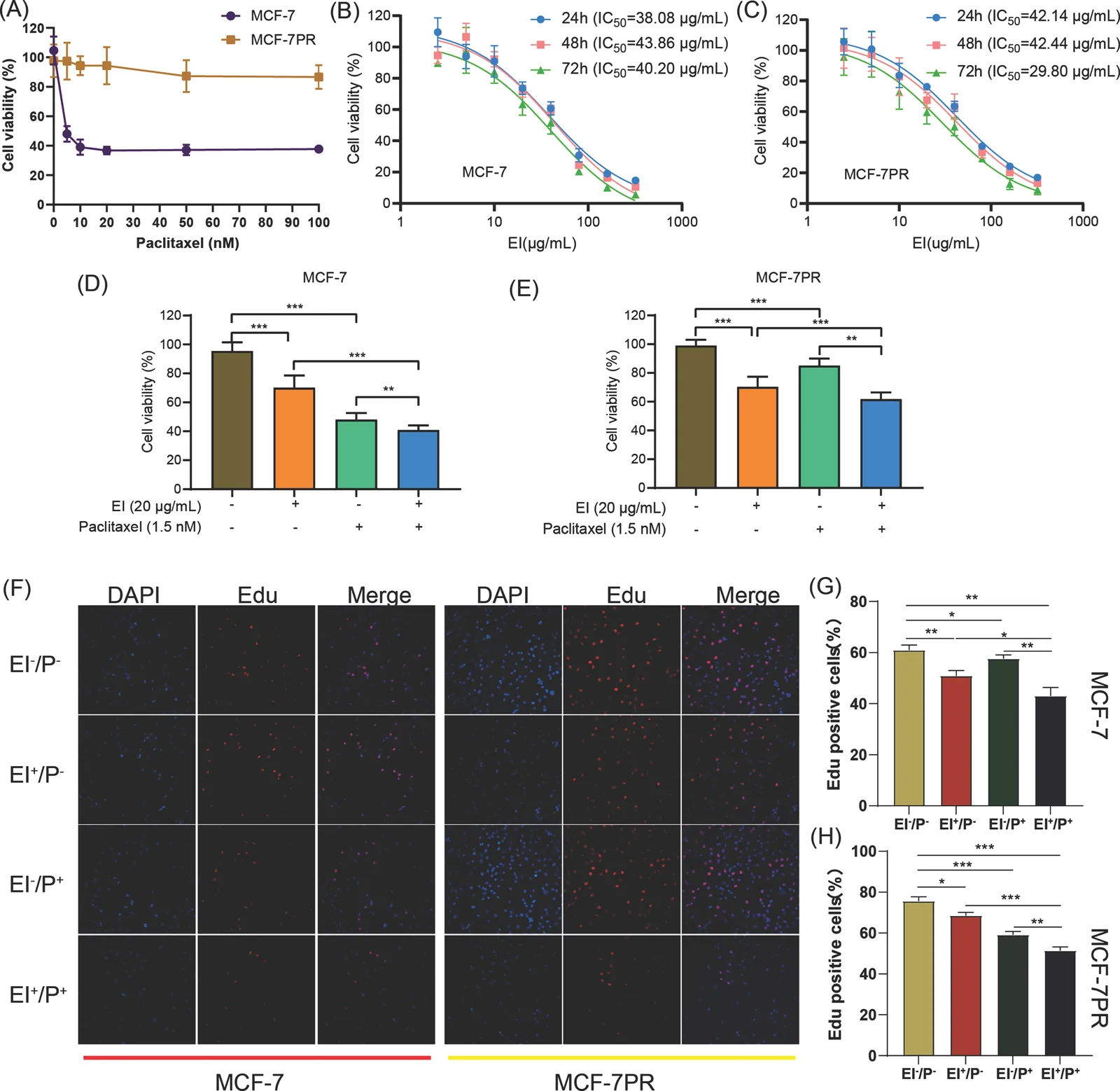
Reversal of PTX resistance in breast cancer by EI. (**A**) Comparison of MCF-7 and MCF-7PR cell viability after 72 hours of PTX induction, estimating cell survival rate. (**B**) Intervention of MCF-7 and (**C**) MCF-7PR cells with varying concentrations of EI for 24, 48, and 72 hours. Cell viability was assessed using the SRB assay to estimate IC_50_ values. (**D**) Percentage of cell survival in MCF-7 cells and (**E**) MCF-7PR cells after pre-treatment with specified concentrations of EI for 2 hours, followed by 72 hours of PTX treatment. (**F**-**H**) Proliferation levels of MCF-7 cells and MCF-7PR cells after pre-treatment with specified concentrations of EI for 2 hours, followed by 72 hours of PTX treatment.

To evaluate the potential of EI in reversing PTX resistance in BC, we established MCF-7 PTX-resistant cell lines. As illustrated in Fig. (**5A**), 100 nM PTX led to 60% cell growth inhibition of MCF-7 cells. However, MCF-7PR cells exhibited resistance to the growth inhibitory properties of 100nM PTX (Fig. **5A**). The data confirmed MCF-7PR cells to have acquired resistance to PTX. Next, the growth inhibitory effects of EI on both PTX-sensitive and resistant cells were assessed. Initially, cytotoxicity assays were conducted on MCF-7 and MCF-PR cells, which were treated with varying concentrations of EI for 24, 48, and 72 hours. The results indicated that increasing concentrations of EI not only significantly suppressed the growth of PTX-sensitive cells, but also inhibited the growth of PTX-resistant cells in a concentration- and time-dependent manner (Fig. **5B**, **C**). To investigate whether EI could enhance the efficacy of PTX and overcome resistance, pre-treatment of MCF-7 and MCF-7PR cells with 20 μg/mL of EI for 2 hours was followed by exposure to PTX for 72 hours. Cell viability was then measured. Our findings revealed that the combination of EI and PTX significantly reduced cell viability compared to PTX alone (Fig. **5D**, **E**). Additionally, EdU staining experiments were performed to examine the inhibitory effects of EI on the proliferative capacity of BC cells. The results of this experiment clearly demonstrated that EI treatment effectively and dose-dependently suppressed the proliferation of MCF-7 and MCF-7PR cells (Fig. **5F**-**H**).

### 3.6 EI Involvement in Tumor Stemness and Chemoresistance by Regulating the AR/RUNX1 Axis

Network pharmacology has demonstrated that AR is a pivotal molecular target for EI, with its interaction displaying the most favorable binding energy among the compound's active components. Western blot analyses showed that treatment with EI significantly downregulated the protein expression levels of both AR and RUNX1 in MCF-7 and its paclitaxel-resistant derivative, MCF-7PR, as depicted in Fig. (**6A**-**E**). Notably, the suppressive effect of EI on the AR/RUNX1 signal was effectively counteracted upon exposure to the AR agonist DHT. Moreover, EI intervention resulted in a marked upregulation of the mRNA expression levels of the cancer stem cell markers, KLF4 and OCT4, within both MCF-7 and MCF-7PR cell lines. Treatment with DHT, however, reversed the overexpression of KLF4 and OCT4 genes induced by EI in these cells, as evidenced in Fig. (**6F**-**I**). These collective findings suggest that EI may exert its influence on attenuating tumor stemness attributes and reversing paclitaxel resistance in BC through the inhibition of the AR/RUNX1 signaling pathway.

## 4 DISCUSSION

PTX, an extensively employed chemotherapeutic agent, holds great significance in the treatment of a diverse range of cancer types, encompassing BC, ovarian cancer, lung cancer, and pancreatic cancer [25-28]. Mechanistically, PTX disrupts the normal process of cellular division in tumor cells, thereby impeding tumor growth and metastasis. Its mode of action involves binding to and stabilizing microtubule proteins, thereby impeding the formation of microtubules essential for tumor cell division, ultimately leading to cellular demise. Regrettably, the development of resistance to PTX represents a significant impediment to the efficacy of BC chemotherapy. Thus, comprehending the intricate molecular mechanisms that underlie chemotherapy resistance assumes paramount importance in the pursuit of novel drug targets. Elucidating the mechanisms of PTX resistance has been a subject of limited exploration, encompassing the activation of the PI3K/Akt and hedgehog/ GSK3β signaling pathways, drug efflux transporters, and mutations in β-tubulin [29]. In order to shed light on the molecular determinants governing the reversal of PTX resistance in BC, we undertook a comprehensive reanalysis of the GSE144113 dataset, leading to the identification of 761 genes exhibiting a compelling association with PTX resistance. This endeavor served as a pivotal foundation for subsequent network pharmacology analyses.

As a novel phytochemical entity, β-elemene demonstrates a wide range of anti-cancer attributes, rendering it a promising candidate for anti-metastatic drug development. Extensive research has consistently attested to its tumor-inhibitory effects and capacity to counteract drug resistance. However, its underlying molecular mechanisms remain incompletely elucidated. Notably, the extensively examined β-elemene conformation has been shown to potentiate apoptosis in prostate cancer when combined with cisplatin, primarily mediated by the modulation of apoptosis-associated genes within neoplastic cells [30]. Clinical trials have further substantiated the efficacy and tolerability of β-elemene in lung cancer therapeutics [31]. Zhou *et al*. [32] have validated the synergistic anti-proliferative properties of a novel combination therapy involving mTOR inhibitors and β-elemene in the context of follicular thyroid cancer. Beyond its cytotoxic effects, β-elemene suppresses BC cell migration and invasion, rendering it a promising candidate for targeted heparanase-focused therapy in breast carcinomas [33]. In the realm of drug resistance, β-elemene alleviates gefitinib resistance in non-small cell lung cancer *via* mettl3-modulated autophagy [34], and opposes 5-fluorouracil resistance in p53-null colorectal cancers through autophagy-induced cell death initiation and cyclin D3-driven cell cycle arrest [35]. In BC, it mitigates chemotherapy resistance by inhibiting exosome-mediated drug resistance spread and fine-tuning microRNA profiles linked to multidrug resistance [36, 37]. In this study, the synergistic inhibitory effects of β-elemene in conjunction with PTX on BC cell proliferation were unveiled, concurrently demonstrating the sensitization of PTX-resistant MCF-7PR cell line, thereby further broadening the prospective applications of β-elemene.

The advancement of high-throughput sequencing technology and network pharmacology has facilitated a systematic exploration of drug action networks and mechanisms, thereby departing from the conventional “one drug, one target” paradigm [38]. For instance, Wang *et al*. [39] integrated network pharmacology analysis and cytokine array screening to identify tumor-associated macrophage/C-X-C chemokine ligand 1 as a pivotal regulatory factor in preventing BC metastasis through Xiaopi Fang, a TCM formulation. Through the integration of chemoinformatics, bioinformatics, and network biology, they successfully predicted the active compounds of Tianfushen oral liquid and validated their therapeutic targets in colorectal cancer [40]. In cancer resistance studies, Wang *et al*. applied network pharmacology approaches to elucidate Caveolin-1 as a critical mediator of resistance to Aidiqing, an anti-cancer drug [41]. Herein, our network analysis revealed 122 candidate targets for the three active constituents present in EI. Subsequently, upon intersecting with PTX resistance-associated genes acquired from the GSE144113 dataset, nine potential targets capable of reversing PTX resistance were identified.

Among the potential targets, TP53, a central hub due to its high degree of centrality in the PPI network, may play a crucial role in overcoming PTX resistance in BC, particularly through the action of β-elemene. As a key tumor suppressor, p53 regulates cell cycle progression, DNA repair, and apoptosis, which are critical in the response to chemotherapy [42]. In addition, IL6, implicated in aggressive disease and poor outcomes [43], is known to promote resistance to endocrine therapy and chemotherapy by enhancing cell survival, angiogenesis, and invasion [44, 45]. CES1, an enzyme involved in drug metabolism, has been associated with the inactivation of chemotherapy agents [46, 47], suggesting that its overexpression might contribute to BC's resistance to chemotherapy. CNR1 and PPARD, involved in cell signaling and lipid metabolism, respectively, can influence the sensitivity of BC cells to chemotherapy and endocrine therapy [48, 49]. Their altered expression may affect programmed cell death and treatment response. CDK1 and CDKN1B, key regulators of the cell cycle, are also implicated in resistance mechanisms [50, 51]. Overexpression of CDK1 can lead to uncontrolled cell division and chemotherapy resistance [52], while loss of CDKN1B function can result in cell cycle dysregulation and resistance to therapy [53].

In recent years, accumulating evidence has highlighted a complex interplay between AR expression and therapeutic resistance in BC, particularly in Triple-negative BC (TNBC). Studies have demonstrated that AR is expressed in up to 50% of TNBC cases [54], and its inhibition has been shown to decrease cancer stem cell populations and tumor initiation, suggesting a potential target for overcoming resistance to chemotherapy. For instance, Liu *et al*. revealed a high expression of AIB1, an amplifier gene in BC, to be correlated with tamoxifen resistance [55]. Their findings have indicated that patients with ER-positive BC who express high levels of AIB1 are more likely to develop TAM resistance, which could be further exacerbated when combined with AR positivity. This implies that co-targeting AR and AIB1 pathways might improve response to endocrine therapy. Moreover, Ali *et al*. discussed the intricate relationship between hypoxia signaling networks and AR activation in BC, where hypoxia promotes resistance to various anticancer drugs, including AR inhibitors [55]. This underscores the importance of considering the tumor microenvironment when devising strategies to overcome treatment resistance. Our research study has introduced novel insights into this landscape by demonstrating that EI, a compound derived from traditional Chinese medicine, exerts its action by inhibiting AR/RUNX1 signaling, thereby reversing paclitaxel resistance in BC cells.

BC chemotherapy resistance implicates an inadequacy in understanding the molecular mechanisms of Chinese medicine components, notably in the AR/RUNX1 signaling axis. Our systematic screening and validation have pinpointed EI's modulation of PTX resistance-related genes, offering fresh insights into addressing this gap. A deeper exploration of EI's multitarget mechanisms is poised to inform more efficacious strategies for reversing drug resistance. With advancements in the next five years, refined knowledge of compounds, like EI, may usher in precision therapies for resistant BC, particularly within personalized medicine contexts where patient-specific AR/RUNX1 profiles could guide tailored use of EI or similar natural agents. Synergizing EI with chemotherapy or targeted treatments to amplify resistance reversal has thus emerged as a pivotal research frontier.

## CONCLUSION

This study has demonstrated EI's efficacy in overcoming PTX resistance in breast cancer by targeting nine resistance-linked genes, confirmed through bioinformatic and experimental analyses. It has revealed a dose- and time-dependent response of both PTX-sensitive and resistant cells, implicating AR/RUNX1 signaling and stemness marker downregulation as central mechanisms. These findings position EI as a promising therapeutic strategy to enhance chemotherapy outcomes in breast cancer patients by precision targeting of resistance pathways.

## Figures and Tables

**Fig. (6):**
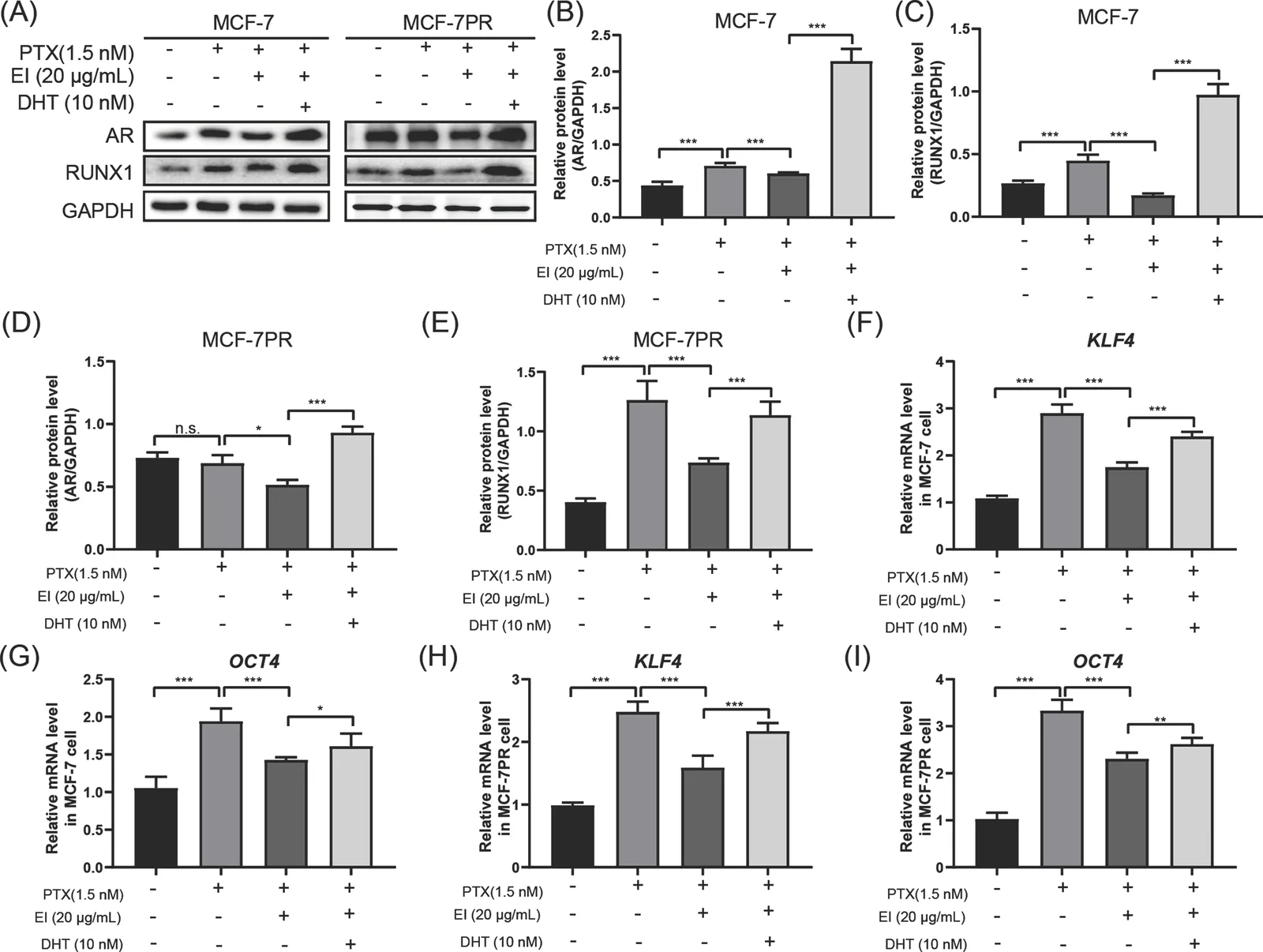
Regulation of tumor stemness markers and chemotherapy resistance by EI *via* the AR/RUNX1 axis. (**A**, **B**) Western blot analysis depicting protein expression levels of AR and RUNX1 in MCF-7 (**A**) and MCF-7PR cells (**B**). (**B**-**E**) Statistical evaluation of AR and RUNX1 protein expression levels in MCF-7 and MCF-7PR cells, where n.s. denotes not significant; *p < 0.05, **p < 0.01, ***p < 0.001. (**F**-**I**) Statistical assessment of mRNA expression levels for the tumor stem cell markers, KLF4 and OCT4, in both MCF-7 and MCF-7PR cells, with significance denoted as *p < 0.05, **p < 0.01, ***p < 0.001.

## Data Availability

The data that support the findings of this study are available from the corresponding authors [X.H.X. and X.B.S.], upon reasonable request.

## LIST OF ABBREVIATIONS

AR: Androgen Receptor
BC: Breast Cancer
CDK1: Cyclin-dependent Kinase 1
CDKN1B: Cyclin-dependent Kinase Inhibitor 1B
CES1: Carboxylesterase 1
CNR1: Cannabinoid Receptor 1
CTD: Comparative Toxicogenomics Database
DHT: Dihydrotestosterone
DMSO: Dimethyl Sulfoxide
ECL: Enhanced Chemiluminescence
EI: Elemene Injection
FBS: Fetal Bovine Serum
IL6: Interleukin-6
PDB: Protein Data Bank
PPARD: Peroxisome Proliferator-activated Receptor Delta
PTX: Paclitaxel
SD: Standard Deviation
SRB: Sulforhodamine B
STP: SwissTargetPrediction
TCM: Traditional Chinese Medicine
TCMSP: Traditional Chinese Medicine Systems Pharmacology Database and Analysis Platform
TP53: Tumor Protein p53
