# Supplementary material for: Elemene Injection Overcomes Paclitaxel Resistance in Breast Cancer through AR/RUNX1 Signal: Network Pharmacology and Experimental Validation
Source: Curr Pharm Des. 2024 Jun 24;30(29):2313–24. doi: 10.2174/0113816128315677240620052444 (PMC11475252; doi:10.2174/0113816128315677240620052444)
Supplement: Supplementary file 1 [file CPD-30-2313_SD1.pdf]

Supplementary Material

Elemene Injection Overcomes Paclitaxel Resistance in Breast Cancer through AR/RUNX1 Signal: Network Pharmacology and Experimental Validation

Xidong Gu[1], Leilai Xu[1], Yuanyuan Fu[1], Shuyao Fan[1], Tianjian Huang[2], Jiangting Yu[2], Jiaying Chen[2], Xinbing Sui[3,*] and Xiaohong Xie[1,*]

[1]*Department of Breast Surgery, The First Affiliated Hospital of Zhejiang Chinese Medical University, Hangzhou 310003, Zhejiang, China;* [2]*Zhejiang Chinese Medical University, Hangzhou 310053, Zhejiang, China;* [3]*School of Pharmacy and Department of Medical Oncology, The Affiliated Hospital of Hangzhou Normal University, Hangzhou 310015, Zhejiang, China*

1873-4286/24 © 2024 Bentham Science Publishers

Table S1. Genes associated with paclitaxel resistance in breast cancer identified by differential expression analysis.

No.	ID	Category	Symbol
1	23067830	Control	
2	23067837	Control	
3	23069176	Control	
4	23069177	Control	
5	23070203	Control	
6	23070259	Control	
7	23070285	Control	
8	23071521	Control	
9	23071880	Control	
10	23071882	Control	
11	23071906	Control	
12	23071908	Control	
13	23071929	Control	
14	23071950	Control	
15	23071971	Control	
16	23071989	Control	
17	23071991	Control	
18	23072026	Control	
19	23072053	Control	
20	23074883	Control	
21	23074889	Control	
22	23074918	Control	
23	23074920	Control	
24	23075519	Control	
25	23075524	Control	
26	23075525	Control	
27	23075526	Control	
28	23075547	Control	
29	23075552	Control	
30	23075561	Control	
31	23075571	Control	
32	23075572	Control	
33	23075575	Control	

No.	ID	Category	Symbol
34	23075610	Control	
35	23075613	Control	
36	23075621	Control	
37	23075624	Control	
38	23075631	Control	
39	23075635	Control	
40	23075636	Control	
41	23075637	Control	
42	23075649	Control	
43	23075651	Control	
44	23075671	Control	
45	23075672	Control	
46	23075690	Control	
47	23075691	Control	
48	23075697	Control	
49	23075702	Control	
50	23075711	Control	
51	23075713	Control	
52	23075714	Control	
53	23075715	Control	
54	23075717	Control	
55	23075718	Control	
56	23075721	Control	
57	23075745	Control	
58	23075783	Control	
59	23075786	Control	
60	23075794	Control	
61	23075795	Control	
62	23075802	Control	
63	23075807	Control	
64	23075808	Control	
65	23075809	Control	
66	23075823	Control	
67	23075825	Control	

No.	ID	Category	Symbol
68	23075843	Control	
69	23075844	Control	
70	23075863	Control	
71	23075870	Control	
72	23075884	Control	
73	23075886	Control	
74	23075887	Control	
75	23075888	Control	
76	23075890	Control	
77	23075891	Control	
78	23075894	Control	
79	23075909	Control	
80	23075944	Control	
81	23075947	Control	
82	23075954	Control	
83	23075961	Control	
84	23075992	Control	
85	23075993	Control	
86	23076017	Control	
87	23076027	Control	
88	23076029	Control	
89	23076030	Control	
90	23076032	Control	
91	23076033	Control	
92	23076039	Control	
93	23076049	Control	
94	23076080	Control	
95	23076083	Control	
96	23076091	Control	
97	23076093	Control	
98	23076100	Control	
99	23076117	Control	
100	23076119	Control	
101	23076138	Control	

No.	ID	Category	Symbol
102	23076155	Control	
103	23076161	Control	
104	23076167	Control	
105	23076178	Control	
106	23076180	Control	
107	23076181	Control	
108	23076182	Control	
109	23076186	Control	
110	23076194	Control	
111	23076195	Control	
112	23076219	Control	
113	23076243	Control	
114	23076246	Control	
115	23076254	Control	
116	23076256	Control	
117	23076263	Control	
118	23076268	Control	
119	23076269	Control	
120	23076270	Control	
121	23076280	Control	
122	23076302	Control	
123	23076303	Control	
124	23076329	Control	
125	23076334	Control	
126	23076344	Control	
127	23076347	Control	
128	23076348	Control	
129	23076350	Control	
130	23076351	Control	
131	23076354	Control	
132	23076362	Control	
133	23076373	Control	
134	23076407	Control	
135	23076410	Control	

No.	ID	Category	Symbol
136	23076414	Control	
137	23076424	Control	
138	23076434	Control	
139	23076461	Control	
140	23076462	Control	
141	23076477	Control	
142	23076481	Control	
143	23076487	Control	
144	23076501	Control	
145	23076502	Control	
146	23076520	Control	
147	23076620	Control	
148	HTA2-neg-47420711_st	normgene->intron	
149	HTA2-neg-47421730_st	normgene->intron	
150	HTA2-pos-2891949_st	normgene->exon	
151	HTA2-pos-2909145_st	normgene->exon	
152	HTA2-pos-2909160_st	normgene->exon	
153	HTA2-pos-2909166_st	normgene->exon	
154	HTA2-pos-2909181_st	normgene->exon	
155	HTA2-pos-2909185_st	normgene->exon	
156	HTA2-pos-2909197_st	normgene->exon	
157	HTA2-pos-2909199_st	normgene->exon	
158	HTA2-pos-3064816_st	normgene->exon	
159	HTA2-pos-3080345_st	normgene->exon	
160	HTA2-pos-3305613_st	normgene->exon	
161	HTA2-pos-3374143_st	normgene->exon	
162	HTA2-pos-3374150_st	normgene->exon	
163	HTA2-pos-3520364_st	normgene->exon	
164	HTA2-pos-3545874_st	normgene->exon	
165	HTA2-pos-3671550_st	normgene->exon	
166	HTA2-pos-PSR02004944.hg.1	normgene->exon	
167	HTA2-pos-PSR06002918.hg.1	normgene->exon	
168	HTA2-pos-PSR06002924.hg.1	normgene->exon	
169	HTA2-pos-PSR06002925.hg.1	normgene->exon	

No.	ID	Category	Symbol
170	HTA2-pos-PSR06002926.hg.1	normgene->exon	
171	HTA2-pos-PSR06002937.hg.1	normgene->exon	
172	HTA2-pos-PSR06002942.hg.1	normgene->exon	
173	HTA2-pos-PSR06002943.hg.1	normgene->exon	
174	HTA2-pos-PSR06002945.hg.1	normgene->exon	
175	HTA2-pos-PSR06002948.hg.1	normgene->exon	
176	HTA2-pos-PSR06007468.hg.1	normgene->exon	
177	HTA2-pos-PSR06007484.hg.1	normgene->exon	
178	HTA2-pos-PSR06007487.hg.1	normgene->exon	
179	HTA2-pos-PSR06007488.hg.1	normgene->exon	
180	HTA2-pos-PSR06017407.hg.1	normgene->exon	
181	HTA2-pos-PSR06017443.hg.1	normgene->exon	
182	HTA2-pos-PSR06017448.hg.1	normgene->exon	
183	HTA2-pos-PSR06035599.hg.1	normgene->exon	
184	HTA2-pos-PSR07002495.hg.1	normgene->exon	
185	HTA2-pos-PSR12025168.hg.1	normgene->exon	
186	HTA2-pos-PSR12026869.hg.1	normgene->exon	
187	HTA2-pos-PSR14004993.hg.1	normgene->exon	
188	HTA2-pos-PSR19006627.hg.1	normgene->exon	
189	HTA2-pos-PSR6_apd_hap1001117.hg.1	normgene->exon	
190	HTA2-pos-PSR6_cox_hap2000465.hg.1	normgene->exon	
191	HTA2-pos-PSR6_cox_hap2000471.hg.1	normgene->exon	
192	HTA2-pos-PSR6_cox_hap2000472.hg.1	normgene->exon	
193	HTA2-pos-PSR6_cox_hap2000473.hg.1	normgene->exon	
194	HTA2-pos-PSR6_cox_hap2000481.hg.1	normgene->exon	
195	HTA2-pos-PSR6_cox_hap2000486.hg.1	normgene->exon	
196	HTA2-pos-PSR6_cox_hap2000487.hg.1	normgene->exon	
197	HTA2-pos-PSR6_cox_hap2000489.hg.1	normgene->exon	
198	HTA2-pos-PSR6_cox_hap2000492.hg.1	normgene->exon	
199	HTA2-pos-PSR6_cox_hap2002754.hg.1	normgene->exon	
200	HTA2-pos-PSR6_cox_hap2002786.hg.1	normgene->exon	
201	HTA2-pos-PSR6_cox_hap2002790.hg.1	normgene->exon	
202	HTA2-pos-PSR6_dbb_hap3000454.hg.1	normgene->exon	
203	HTA2-pos-PSR6_dbb_hap3000460.hg.1	normgene->exon	

No.	ID	Category	Symbol
204	HTA2-pos-PSR6_dbb_hap3000461.hg.1	normgene->exon	
205	HTA2-pos-PSR6_dbb_hap3000462.hg.1	normgene->exon	
206	HTA2-pos-PSR6_dbb_hap3000470.hg.1	normgene->exon	
207	HTA2-pos-PSR6_dbb_hap3000475.hg.1	normgene->exon	
208	HTA2-pos-PSR6_dbb_hap3000476.hg.1	normgene->exon	
209	HTA2-pos-PSR6_dbb_hap3000478.hg.1	normgene->exon	
210	HTA2-pos-PSR6_dbb_hap3000481.hg.1	normgene->exon	
211	HTA2-pos-PSR6_dbb_hap3002667.hg.1	normgene->exon	
212	HTA2-pos-PSR6_dbb_hap3002699.hg.1	normgene->exon	
213	HTA2-pos-PSR6_dbb_hap3002703.hg.1	normgene->exon	
214	HTA2-pos-PSR6_mann_hap4000445.hg.1	normgene->exon	
215	HTA2-pos-PSR6_mann_hap4000451.hg.1	normgene->exon	
216	HTA2-pos-PSR6_mann_hap4000452.hg.1	normgene->exon	
217	HTA2-pos-PSR6_mann_hap4000453.hg.1	normgene->exon	
218	HTA2-pos-PSR6_mann_hap4000461.hg.1	normgene->exon	
219	HTA2-pos-PSR6_mann_hap4000466.hg.1	normgene->exon	
220	HTA2-pos-PSR6_mann_hap4000467.hg.1	normgene->exon	
221	HTA2-pos-PSR6_mann_hap4000469.hg.1	normgene->exon	
222	HTA2-pos-PSR6_mann_hap4000472.hg.1	normgene->exon	
223	HTA2-pos-PSR6_mann_hap4002282.hg.1	normgene->exon	
224	HTA2-pos-PSR6_mann_hap4002314.hg.1	normgene->exon	
225	HTA2-pos-PSR6_mann_hap4002318.hg.1	normgene->exon	
226	HTA2-pos-PSR6_mcf_hap5000354.hg.1	normgene->exon	
227	HTA2-pos-PSR6_mcf_hap5000360.hg.1	normgene->exon	
228	HTA2-pos-PSR6_mcf_hap5000361.hg.1	normgene->exon	
229	HTA2-pos-PSR6_mcf_hap5000362.hg.1	normgene->exon	
230	HTA2-pos-PSR6_mcf_hap5000370.hg.1	normgene->exon	
231	HTA2-pos-PSR6_mcf_hap5000375.hg.1	normgene->exon	
232	HTA2-pos-PSR6_mcf_hap5000376.hg.1	normgene->exon	
233	HTA2-pos-PSR6_mcf_hap5000378.hg.1	normgene->exon	
234	HTA2-pos-PSR6_mcf_hap5000381.hg.1	normgene->exon	
235	HTA2-pos-PSR6_mcf_hap5002424.hg.1	normgene->exon	
236	HTA2-pos-PSR6_mcf_hap5002456.hg.1	normgene->exon	
237	HTA2-pos-PSR6_mcf_hap5002460.hg.1	normgene->exon	

No.	ID	Category	Symbol
238	HTA2-pos-PSR6_qbl_hap6000451.hg.1	normgene->exon	
239	HTA2-pos-PSR6_qbl_hap6000457.hg.1	normgene->exon	
240	HTA2-pos-PSR6_qbl_hap6000458.hg.1	normgene->exon	
241	HTA2-pos-PSR6_qbl_hap6000459.hg.1	normgene->exon	
242	HTA2-pos-PSR6_qbl_hap6000467.hg.1	normgene->exon	
243	HTA2-pos-PSR6_qbl_hap6000472.hg.1	normgene->exon	
244	HTA2-pos-PSR6_qbl_hap6000473.hg.1	normgene->exon	
245	HTA2-pos-PSR6_qbl_hap6000475.hg.1	normgene->exon	
246	HTA2-pos-PSR6_qbl_hap6000478.hg.1	normgene->exon	
247	HTA2-pos-PSR6_qbl_hap6002735.hg.1	normgene->exon	
248	HTA2-pos-PSR6_qbl_hap6002767.hg.1	normgene->exon	
249	HTA2-pos-PSR6_qbl_hap6002771.hg.1	normgene->exon	
250	HTA2-pos-PSR6_ssto_hap7000448.hg.1	normgene->exon	
251	HTA2-pos-PSR6_ssto_hap7000454.hg.1	normgene->exon	
252	HTA2-pos-PSR6_ssto_hap7000455.hg.1	normgene->exon	
253	HTA2-pos-PSR6_ssto_hap7000456.hg.1	normgene->exon	
254	HTA2-pos-PSR6_ssto_hap7000464.hg.1	normgene->exon	
255	HTA2-pos-PSR6_ssto_hap7000469.hg.1	normgene->exon	
256	HTA2-pos-PSR6_ssto_hap7000470.hg.1	normgene->exon	
257	HTA2-pos-PSR6_ssto_hap7000472.hg.1	normgene->exon	
258	HTA2-pos-PSR6_ssto_hap7000475.hg.1	normgene->exon	
259	HTA2-pos-PSR6_ssto_hap7002423.hg.1	normgene->exon	
260	HTA2-pos-PSR6_ssto_hap7002455.hg.1	normgene->exon	
261	HTA2-pos-PSR6_ssto_hap7002459.hg.1	normgene->exon	
355	TC0200011219.hg.1	Homo sapiens atypical chemokine receptor 3	ACKR3
892	TC1700008254.hg.1	Homo sapiens acyl-CoA synthetase family member 2	ACSF2
725	TC1000011370.hg.1	Homo sapiens actin, alpha 2, smooth muscle, aorta	ACTA2
501	TC0500010822.hg.1	Homo sapiens actin, beta-like 2	ACTBL2
374	TC0200014697.hg.1	Homo sapiens activin A receptor, type I	ACVR1
357	TC0200011688.hg.1	Homo sapiens ADAM metallopeptidase domain 17	ADAM17
735	TC1000012516.hg.1	Homo sapiens aldo-keto reductase family 1, member C2	AKR1C2
977	TC1900011778.hg.1	Homo sapiens aldehyde dehydrogenase 16 family, member A1	ALDH16A1
873	TC1500010800.hg.1	Homo sapiens aldehyde dehydrogenase 1 family, member A3	ALDH1A3
519	TC0600007198.hg.1	Homo sapiens aldehyde dehydrogenase 5 family, member A1	ALDH5A1

No.	ID	Category	Symbol
769	TC1100013226.hg.1	Homo sapiens ALG9, alpha-1,2-mannosyltransferase	ALG9
337	TC0200008047.hg.1	Homo sapiens Alstrom syndrome protein 1	ALMS1
709	TC1000007986.hg.1	Homo sapiens anaphase promoting complex subunit 16	ANAPC16
660	TC0800011445.hg.1	Homo sapiens angiopoietin 1	ANGPT1
495	TC0500010169.hg.1	Homo sapiens ANKH inorganic pyrophosphate transport regulator	ANKH
931	TC1800009215.hg.1	Homo sapiens ankyrin repeat domain 12	ANKRD12
540	TC0600007774.hg.1	Homo sapiens ankyrin repeat and sterile alpha motif domain containing 1A	ANKS1A
736	TC1000012551.hg.1	Homo sapiens annexin A8	ANXA8
733	TC1000012454.hg.1	Homo sapiens annexin A8-like 1	ANXA8L1
351	TC0200010421.hg.1	Homo sapiens aldehyde oxidase 1	AOX1
685	TC0X00007493.hg.1	Homo sapiens androgen receptor	AR
480	TC0400012917.hg.1	Homo sapiens ArfGAP with RhoGAP domain, ankyrin repeat and PH domain 2	ARAP2
761	TC1100012269.hg.1	Homo sapiens Rho GTPase activating protein 20	ARHGAP20
462	TC0400008030.hg.1	Homo sapiens Rho GTPase activating protein 24	ARHGAP24
614	TC0700006737.hg.1	Homo sapiens ADP-ribosylation factor-like 4A	ARL4A
697	TC0X00010369.hg.1	Homo sapiens armadillo repeat containing, X-linked 2	ARMCX2
314	TC0100016831.hg.1	Homo sapiens abnormal spindle microtubule assembly	ASPM
885	TC1700007469.hg.1	Homo sapiens ATPase family, AAA domain containing 5	ATAD5
602	TC0600014098.hg.1	Homo sapiens alpha tubulin acetyltransferase 1	ATAT1
416	TC0300009651.hg.1	Homo sapiens ATPase, class VI, type 11B	ATP11B
961	TC1900010782.hg.1	Homo sapiens ATPase, Na+/K+ transporting, alpha 3 polypeptide	ATP1A3
282	TC0100010543.hg.1	Homo sapiens ATPase, Na+/K+ transporting, beta 1 polypeptide	ATP1B1
874	TC1600008646.hg.1	Homo sapiens ATPase, Ca++ transporting, type 2C, member 2	ATP2C2
866	TC1400010760.hg.1	Homo sapiens ATPase, H+ transporting, lysosomal 34kDa, V1 subunit D	ATP6V1D
953	TC1900009186.hg.1	Homo sapiens ATPase, aminophospholipid transporter, class I, type 8B, member 3	ATP8B3
564	TC0600010921.hg.1	Homo sapiens ataxin 1	ATXN1
900	TC1700009679.hg.1	Homo sapiens aurora kinase B	AURKB
617	TC0700007905.hg.1	Homo sapiens autism susceptibility candidate 2	AUTS2
271	TC0100007705.hg.1	Homo sapiens antizyme inhibitor 2	AZIN2
937	TC1900007344.hg.1	Homo sapiens UDP-GlcNAc:betaGal beta-1,3-N-acetylglucosaminyltransferase 3	B3GNT3
417	TC0300009661.hg.1	Homo sapiens UDP-GlcNAc:betaGal beta-1,3-N-acetylglucosaminyltransferase 5	B3GNT5

No.	ID	Category	Symbol
755	TC1100011285.hg.1	Homo sapiens beta-1,4-glucuronyltransferase 1	B4GAT1
878	TC1600010409.hg.1	Homo sapiens Bardet-Biedl syndrome 2	BBS2
965	TC1900011098.hg.1	Homo sapiens branched chain amino-acid transaminase 2, mitochondrial	BCAT2
973	TC1900011744.hg.1	Homo sapiens branched chain keto acid dehydrogenase E1, alpha polypeptide	BCKDHA
751	TC1100010410.hg.1	Homo sapiens brain-derived neurotrophic factor	BDNF
691	TC0X00008785.hg.1	Homo sapiens biglycan	BGN
792	TC1200010182.hg.1	Homo sapiens basic helix-loop-helix family, member e41	BHLHE41
767	TC1100013088.hg.1	Homo sapiens baculoviral IAP repeat containing 2	BIRC2
976	TC1900011767.hg.1	Homo sapiens biogenesis of lysosomal organelles complex-1, subunit 3	BLOC1S3
846	TC1400009214.hg.1	Homo sapiens bone morphogenetic protein 4	BMP4
645	TC0800007080.hg.1	Homo sapiens BCL2/adenovirus E1B 19kDa interacting protein 3-like	BNIP3L
368	TC0200013095.hg.1	Homo sapiens bolA family member 3	BOLA3
828	TC1400006913.hg.1	Homo sapiens breast cancer metastasis-suppressor 1-like	BRMS1L
854	TC1400009732.hg.1	Homo sapiens chromosome 14 open reading frame 1	C14orf1
891	TC1700007982.hg.1	Homo sapiens chromosome 17 open reading frame 53	C17orf53
968	TC1900011221.hg.1	Homo sapiens chromosome 19 open reading frame 48	C19orf48
908	TC1700010856.hg.1	Homo sapiens complement component 1, q subcomponent-like 1	C1QL1
894	TC1700009079.hg.1	Homo sapiens C1q and tumor necrosis factor related protein 1	C1QTNF1
816	TC1200012744.hg.1	Homo sapiens complement component 1, r subcomponent	C1R
811	TC1200012592.hg.1	Homo sapiens complement component 1, s subcomponent	C1S
381	TC0200016589.hg.1	Homo sapiens chromosome 2 open reading frame 69	C2orf69
535	TC0600007617.hg.1	Homo sapiens chromosome 6 open reading frame 48	C6orf48
871	TC1500009709.hg.1	Homo sapiens carbonic anhydrase XII	CA12
950	TC1900008826.hg.1	Homo sapiens calcium channel, voltage-dependent, gamma subunit 7	CACNG7
472	TC0400011643.hg.1	Homo sapiens calcium/calmodulin-dependent protein kinase II delta	CAMK2D
897	TC1700009492.hg.1	Homo sapiens calcium/calmodulin-dependent protein kinase kinase 1, alpha	CAMKK1
768	TC1100013222.hg.1	Homo sapiens caspase recruitment domain family, member 16	CARD16
511	TC0500013204.hg.1	Homo sapiens calpastatin	CAST
295	TC0100012849.hg.1	Homo sapiens castor zinc finger 1	CASZ1
1011	TC2200008734.hg.1	Homo sapiens chromobox homolog 7	CBX7
578	TC0600011697.hg.1	Homo sapiens coiled-coil domain containing 167	CCDC167
730	TC1000011892.hg.1	Homo sapiens coiled-coil domain containing 186	CCDC186
429	TC0300012186.hg.1	Homo sapiens coiled-coil domain containing 58	CCDC58

No.	ID	Category	Symbol
771	TC1200006555.hg.1	Homo sapiens cyclin D2	CCND2
492	TC0500009319.hg.1	Homo sapiens cyclin G1	CCNG1
479	TC0400012818.hg.1	Homo sapiens cyclin G2	CCNG2
718	TC1000008529.hg.1	Homo sapiens cyclin J	CCNJ
839	TC1400008173.hg.1	Homo sapiens cyclin K	CCNK
551	TC0600008539.hg.1	Homo sapiens CD109 molecule	CD109
916	TC1700012191.hg.1	Homo sapiens CD68 molecule	CD68
955	TC1900009439.hg.1	Homo sapiens CD70 molecule	CD70
516	TC0600007012.hg.1	Homo sapiens CD83 molecule	CD83
272	TC0100008057.hg.1	Homo sapiens cell division cycle 20	CDC20
644	TC0800007065.hg.1	Homo sapiens cell division cycle associated 2	CDCA2
790	TC1200009759.hg.1	Homo sapiens cell division cycle associated 3	CDCA3
706	TC1000007748.hg.1	Homo sapiens cyclin-dependent kinase 1	CDK1
593	TC0600012839.hg.1	Homo sapiens cyclin-dependent kinase 19	CDK19
634	TC0700011785.hg.1	Homo sapiens cyclin-dependent kinase 6	CDK6
518	TC0600007143.hg.1	Homo sapiens CDK5 regulatory subunit associated protein 1-like 1	CDKAL1
831	TC1400007201.hg.1	Homo sapiens cyclin-dependent kinase inhibitor 3	CDKN3
471	TC0400011477.hg.1	Homo sapiens centromere protein E, 312kDa	CENPE
287	TC0100011581.hg.1	Homo sapiens centromere protein F, 350/400kDa	CENPF
312	TC0100016441.hg.1	Homo sapiens centromere protein L	CENPL
330	TC0200006977.hg.1	Homo sapiens centromere protein O	CENPO
924	TC1800006749.hg.1	Homo sapiens centrosomal protein 192kDa	CEP192
717	TC1000008482.hg.1	Homo sapiens centrosomal protein 55kDa	CEP55
1012	TC2200009028.hg.1	Homo sapiens ceramide kinase	CERK
877	TC1600010394.hg.1	Homo sapiens carboxylesterase 1	CES1
284	TC0100011064.hg.1	Homo sapiens complement factor H	CFH
814	TC1200012698.hg.1	Homo sapiens choline phosphotransferase 1	CHPT1
422	TC0300010676.hg.1	Homo sapiens cytoplasmic linker associated protein 2	CLASP2
762	TC1100012615.hg.1	Homo sapiens CXADR-like membrane protein	CLMP
393	TC0300006961.hg.1	Homo sapiens CKLF-like MARVEL transmembrane domain containing 8	CMTM8
546	TC0600007869.hg.1	Homo sapiens cap methyltransferase 1	CMTR1
740	TC1100008069.hg.1	Homo sapiens cornichon family AMPA receptor auxiliary protein 2	CNIH2
324	TC0100018349.hg.1	Homo sapiens cornichon family AMPA receptor auxiliary protein 3	CNIH3
289	TC0100011746.hg.1	Homo sapiens cornichon family AMPA receptor auxiliary protein 4	CNIH4

No.	ID	Category	Symbol
367	TC0200012933.hg.1	Homo sapiens cannabinoid receptor interacting protein 1	CNRIP1
918	TC1700012260.hg.1	Homo sapiens CoA synthase	COASY
910	TC1700011210.hg.1	Homo sapiens coilin	COIL
621	TC0700009134.hg.1	Homo sapiens carboxypeptidase A4	CPA4
464	TC0400009223.hg.1	Homo sapiens carboxypeptidase E	CPE
620	TC0700008928.hg.1	Homo sapiens cadherin-like and PC-esterase domain containing 1	CPED1
793	TC1200010397.hg.1	Homo sapiens copine VIII	CPNE8
648	TC0800008300.hg.1	Homo sapiens carboxypeptidase Q	CPQ
311	TC0100016018.hg.1	Homo sapiens cellular retinoic acid binding protein 2	CRABP2
616	TC0700007034.hg.1	Homo sapiens cAMP responsive element binding protein 5	CREB5
494	TC0500009488.hg.1	Homo sapiens CREB3 regulatory factor	CREBRF
842	TC1400008486.hg.1	Homo sapiens cysteine-rich protein 2	CRIP2
647	TC0800007995.hg.1	Homo sapiens cysteine-rich secretory protein LCCL domain containing 1	CRISPLD1
675	TC0900010933.hg.1	Homo sapiens cathepsin V	CTSV
996	TC2000009522.hg.1	Homo sapiens cytochrome P450, family 24, subfamily A, polypeptide 1	CYP24A1
956	TC1900009896.hg.1	Homo sapiens cytochrome P450, family 4, subfamily F, polypeptide 11	CYP4F11
666	TC0900007167.hg.1	Homo sapiens DDB1 and CUL4 associated factor 10	DCAF10
822	TC1300008609.hg.1	Homo sapiens doublecortin-like kinase 1	DCLK1
747	TC1100009450.hg.1	Homo sapiens decapping enzyme, scavenger	DCPS
529	TC0600007552.hg.1	Homo sapiens discoidin domain receptor tyrosine kinase 1	DDR1
868	TC1500007612.hg.1	Homo sapiens DIS3 like exosome 3-5 exoribonuclease	DIS3L
705	TC1000007641.hg.1	Homo sapiens dickkopf WNT signaling pathway inhibitor 1	DKK1
347	TC0200009967.hg.1	Homo sapiens distal-less homeobox 1	DLX1
695	TC0X00009341.hg.1	Homo sapiens dystrophin	DMD
384	TC0200016647.hg.1	Homo sapiens dpy-30 histone methyltransferase complex regulatory subunit	DPY30
306	TC0100015023.hg.1	Homo sapiens dihydropyrimidine dehydrogenase	DPYD
927	TC1800008385.hg.1	Homo sapiens desmocollin 3	DSC3
929	TC1800008952.hg.1	Homo sapiens dermatan sulfate epimerase-like	DSEL
513	TC0600006869.hg.1	Homo sapiens desmoplakin	DSP
925	TC1800007059.hg.1	Homo sapiens dystrobrevin, alpha	DTNA
563	TC0600010901.hg.1	Homo sapiens dystrobrevin binding protein 1	DTNBP1
510	TC0500012842.hg.1	Homo sapiens dual specificity phosphatase 1	DUSP1
319	TC0100017420.hg.1	Homo sapiens dual specificity phosphatase 10	DUSP10

No.	ID	Category	Symbol
803	TC1200011470.hg.1	Homo sapiens dual specificity phosphatase 6	DUSP6
732	TC1000012399.hg.1	Homo sapiens enoyl CoA hydratase, short chain, 1, mitochondrial	ECHS1
514	TC0600006967.hg.1	Homo sapiens endothelin 1	EDN1
754	TC1100011257.hg.1	Homo sapiens EGF containing fibulin-like extracellular matrix protein 2	EFEMP2
321	TC0100017730.hg.1	Homo sapiens egl-9 family hypoxia-inducible factor 1	EGLN1
737	TC1100007262.hg.1	Homo sapiens ets homologous factor	EHF
409	TC0300009167.hg.1	Homo sapiens eukaryotic translation initiation factor 2A, 65kDa	EIF2A
560	TC0600010802.hg.1	Homo sapiens ELOVL fatty acid elongase 2	ELOVL2
926	TC1800007863.hg.1	Homo sapiens enolase superfamily member 1	ENOSF1
555	TC0600009459.hg.1	Homo sapiens ectonucleotide pyrophosphatase/phosphodiesterase 1	ENPP1
336	TC0200007458.hg.1	Homo sapiens endothelial PAS domain protein 1	EPAS1
505	TC0500011712.hg.1	Homo sapiens erythrocyte membrane protein band 4.1 like 4A	EPB41L4A
678	TC0900011160.hg.1	Homo sapiens erythrocyte membrane protein band 4.1 like 4B	EPB41L4B
639	TC0700013356.hg.1	Homo sapiens ependymin related 1	EPDR1
297	TC0100013028.hg.1	Homo sapiens EPH receptor A2	EPHA2
331	TC0200007035.hg.1	Homo sapiens ethanolaminephosphotransferase 1	EPT1
721	TC1000010602.hg.1	Homo sapiens excision repair cross-complementation group 6	ERCC6
1002	TC2100008143.hg.1	Homo sapiens v-ets avian erythroblastosis virus E26 oncogene homolog	ERG
671	TC0900009470.hg.1	Homo sapiens endoplasmic reticulum metallopeptidase 1	ERMP1
316	TC0100017018.hg.1	Homo sapiens ethanolamine kinase 2	ETNK2
624	TC0700010321.hg.1	Homo sapiens ets variant 1	ETV1
840	TC1400008193.hg.1	Homo sapiens Enah/Vasp-like	EVL
293	TC0100012172.hg.1	Homo sapiens exonuclease 1	EXO1
960	TC1900010746.hg.1	Homo sapiens exosome component 5	EXOSC5
765	TC1100013022.hg.1	Homo sapiens fatty acid desaturase 2	FADS2
406	TC0300008952.hg.1	Homo sapiens Fas apoptotic inhibitory molecule	FAIM
896	TC1700009318.hg.1	Homo sapiens family with sequence similarity 101, member B	FAM101B
278	TC0100009324.hg.1	Homo sapiens family with sequence similarity 102, member B	FAM102B
739	TC1100007728.hg.1	Homo sapiens family with sequence similarity 111, member A	FAM111A
738	TC1100007727.hg.1	Homo sapiens family with sequence similarity 111, member B	FAM111B
352	TC0200010502.hg.1	Homo sapiens family with sequence similarity 117, member B	FAM117B
401	TC0300008551.hg.1	Homo sapiens family with sequence similarity 162, member A	FAM162A
475	TC0400012245.hg.1	Homo sapiens family with sequence similarity 198, member B	FAM198B
420	TC0300009944.hg.1	Homo sapiens family with sequence similarity 43, member A	FAM43A

No.	ID	Category	Symbol
880	TC1700006464.hg.1	Homo sapiens family with sequence similarity 57, member A	FAM57A
986	TC2000007341.hg.1	Homo sapiens family with sequence similarity 83, member D	FAM83D
365	TC0200012219.hg.1	Homo sapiens family with sequence similarity 98, member A	FAM98A
1006	TC2200007620.hg.1	Homo sapiens fibulin 1	FBLN1
978	TC1900011946.hg.1	Homo sapiens F-box protein 27	FBXO27
664	TC0800011683.hg.1	Homo sapiens F-box protein 32	FBXO32
659	TC0800011243.hg.1	Homo sapiens F-box protein 43	FBXO43
264	TC0100006861.hg.1	Homo sapiens F-box protein 44	FBXO44
690	TC0X00008526.hg.1	Homo sapiens four and a half LIM domains 1	FHL1
552	TC0600009102.hg.1	Homo sapiens FIG4 phosphoinositide 5-phosphatase	FIG4
901	TC1700009969.hg.1	Homo sapiens flightless I actin binding protein	FLII
398	TC0300007596.hg.1	Homo sapiens filamin B, beta	FLNB
571	TC0600011381.hg.1	Homo sapiens flotillin 1	FLOT1
991	TC2000008445.hg.1	Homo sapiens fibronectin leucine rich transmembrane protein 3	FLRT3
346	TC0200009680.hg.1	Homo sapiens formin-like 2	FMNL2
463	TC0400009137.hg.1	Homo sapiens folliculin interacting protein 2	FNIP2
863	TC1400010622.hg.1	Homo sapiens farnesyltransferase, CAAX box, beta	FNTB
992	TC2000008636.hg.1	Homo sapiens forkhead box A2	FOXA2
789	TC1200009621.hg.1	Homo sapiens forkhead box M1	FOXM1
824	TC1300008688.hg.1	Homo sapiens forkhead box O1	FOXO1
1010	TC2200008610.hg.1	Homo sapiens FAD-dependent oxidoreductase domain containing 2	FOXRED2
829	TC1400007145.hg.1	Homo sapiens FERM domain containing 6	FRMD6
487	TC0500007376.hg.1	Homo sapiens follistatin	FST
299	TC0100013305.hg.1	Homo sapiens fucosidase, alpha-L- 1, tissue	FUCA1
942	TC1900007836.hg.1	Homo sapiens FXYD domain containing ion transport regulator 3	FXYD3
499	TC0500010553.hg.1	Homo sapiens FYN binding protein	FYB
364	TC0200012161.hg.1	Homo sapiens polypeptide N-acetylgalactosaminyltransferase 14	GALNT14
465	TC0400009322.hg.1	Homo sapiens polypeptide N-acetylgalactosaminyltransferase 7	GALNT7
928	TC1800008418.hg.1	Homo sapiens GRB2 associated, regulator of MAPK1	GAREM
1001	TC2100008000.hg.1	Homo sapiens phosphoribosylglycinamide formyltransferase, phosphoribosylglycinamide synthetase, phosphoribosylaminoimidazole synthetase	GART
782	TC1200008597.hg.1	Homo sapiens growth arrest-specific 2 like 3	GAS2L3
635	TC0700011982.hg.1	Homo sapiens GATS, stromal antigen 3 opposite strand	GATS
305	TC0100014852.hg.1	Homo sapiens guanylate binding protein 1, interferon-inducible	GBP1

No.	ID	Category	Symbol
326	TC0100018451.hg.1	Homo sapiens guanylate binding protein 2, interferon-inducible	GBP2
938	TC1900007384.hg.1	Homo sapiens growth differentiation factor 15	GDF15
485	TC0500007258.hg.1	Homo sapiens growth hormone receptor	GHR
881	TC1700006860.hg.1	Homo sapiens glucagon-like peptide 2 receptor	GLP2R
805	TC1200011752.hg.1	Homo sapiens glycosyltransferase 8 domain containing 2	GLT8D2
313	TC0100016625.hg.1	Homo sapiens glutamate-ammonia ligase	GLUL
827	TC1400006732.hg.1	Homo sapiens guanosine monophosphate reductase 2	GMPR2
804	TC1200011711.hg.1	Homo sapiens N-acetylglucosamine-1-phosphate transferase, alpha and beta subunits	GNPTAB
729	TC1000011839.hg.1	Homo sapiens glycerol-3-phosphate acyltransferase, mitochondrial	GPAM
820	TC1300007718.hg.1	Homo sapiens glypican 6	GPC6
377	TC0200016419.hg.1	Homo sapiens GPN-loop GTPase 1	GPN1
806	TC1200011908.hg.1	Homo sapiens GPN-loop GTPase 3	GPN3
375	TC0200015514.hg.1	Homo sapiens G protein-coupled receptor 1	GPR1
268	TC0100007512.hg.1	Homo sapiens G protein-coupled receptor 3	GPR3
590	TC0600012625.hg.1	Homo sapiens G protein-coupled receptor 63	GPR63
489	TC0500008539.hg.1	Homo sapiens GRAM domain containing 3	GRAMD3
427	TC0300012132.hg.1	Homo sapiens glycogen synthase kinase 3 beta	GSK3B
323	TC0100018261.hg.1	Homo sapiens glutathione S-transferase mu 4	GSTM4
631	TC0700011344.hg.1	Homo sapiens glucuronidase, beta	GUSB
629	TC0700010934.hg.1	Homo sapiens H2A histone family, member V	H2AFV
663	TC0800011646.hg.1	Homo sapiens hyaluronan synthase 2	HAS2
943	TC1900007868.hg.1	Homo sapiens HAUS augmin-like complex, subunit 5	HAUS5
957	TC1900009943.hg.1	Homo sapiens HAUS augmin-like complex, subunit 8	HAUS8
785	TC1200008671.hg.1	Homo sapiens host cell factor C2	HCFC2
432	TC0300012238.hg.1	Homo sapiens heart development protein with EGF-like domains 1	HEG1
470	TC0400011207.hg.1	Homo sapiens helicase, POLQ-like	HELQ
526	TC0600007374.hg.1	Homo sapiens histone cluster 1, H2ai	HIST1H2AI
522	TC0600007282.hg.1	Homo sapiens histone cluster 1, H2bf	HIST1H2BF
523	TC0600007290.hg.1	Homo sapiens histone cluster 1, H2bh	HIST1H2BH
524	TC0600007293.hg.1	Homo sapiens histone cluster 1, H2bi	HIST1H2BI
570	TC0600011224.hg.1	Homo sapiens histone cluster 1, H2bl	HIST1H2BL
567	TC0600011135.hg.1	Homo sapiens histone cluster 1, H3d	HIST1H3D
568	TC0600011142.hg.1	Homo sapiens histone cluster 1, H3g	HIST1H3G
339	TC0200008099.hg.1	Homo sapiens hexokinase 2	HK2

No.	ID	Category	Symbol
611	TC0600014257.hg.1	Homo sapiens major histocompatibility complex, class I, C	HLA-C
537	TC0600007748.hg.1	Homo sapiens high mobility group AT-hook 1	HMGA1
864	TC1400010716.hg.1	Homo sapiens homeobox and leucine zipper encoding	HOMEZ
627	TC0700010564.hg.1	Homo sapiens homeobox A10	HOXA10
626	TC0700010558.hg.1	Homo sapiens homeobox A2	HOXA2
533	TC0600007613.hg.1	Homo sapiens heat shock 70kDa protein 1A	HSPA1A
833	TC1400007443.hg.1	Homo sapiens heat shock 70kDa protein 2	HSPA2
746	TC1100009433.hg.1	Homo sapiens hydrolethalus syndrome 1	HYLS1
935	TC1900006977.hg.1	Homo sapiens intercellular adhesion molecule 1	ICAM1
720	TC1000009536.hg.1	Homo sapiens isopentenyl-diphosphate delta isomerase 1	IDI1
890	TC1700007931.hg.1	Homo sapiens interferon-induced protein 35	IFI35
277	TC0100008815.hg.1	Homo sapiens interferon-induced protein 44-like	IFI44L
763	TC1100012948.hg.1	Homo sapiens interferon induced transmembrane protein 2	IFITM2
630	TC0700010965.hg.1	Homo sapiens insulin-like growth factor binding protein 3	IGFBP3
778	TC1200007693.hg.1	Homo sapiens insulin-like growth factor binding protein 6	IGFBP6
373	TC0200013916.hg.1	Homo sapiens interleukin 1, beta	IL1B
615	TC0700006890.hg.1	Homo sapiens interleukin 6	IL6
484	TC0500007138.hg.1	Homo sapiens interleukin 7 receptor	IL7R
628	TC0700010854.hg.1	Homo sapiens inhibin, beta A	INHBA
865	TC1400010722.hg.1	Homo sapiens importin 4	IPO4
301	TC0100014032.hg.1	Homo sapiens intracisternal A particle-promoted polypeptide	IPP
454	TC0300014043.hg.1	Homo sapiens ISY1 splicing factor homolog	ISY1
455	TC0300014047.hg.1	Homo sapiens ISY1-RAB43 readthrough	ISY1-RAB43
348	TC0200009973.hg.1	Homo sapiens integrin, alpha 6	ITGA6
431	TC0300012233.hg.1	Homo sapiens integrin, beta 5	ITGB5
936	TC1900007096.hg.1	Homo sapiens jun B proto-oncogene	JUNB
995	TC2000009448.hg.1	Homo sapiens potassium channel, voltage gated modifier subfamily G, member 1	KCNG1
343	TC0200008464.hg.1	Homo sapiens Kv channel interacting protein 3, calsenilin	KCNIP3
288	TC0100011592.hg.1	Homo sapiens potassium channel tetramerization domain containing 3	KCTD3
426	TC0300011943.hg.1	Homo sapiens KIAA1524	KIAA1524
716	TC1000008471.hg.1	Homo sapiens kinesin family member 11	KIF11
752	TC1100010418.hg.1	Homo sapiens kinesin family member 18A	KIF18A
715	TC1000008406.hg.1	Homo sapiens kinesin family member 20B	KIF20B
273	TC0100008101.hg.1	Homo sapiens kinesin family member 2C	KIF2C

No.	ID	Category	Symbol
361	TC0200012022.hg.1	Homo sapiens kinesin family member 3C	KIF3C
802	TC1200011460.hg.1	Homo sapiens KIT ligand	KITLG
684	TC0X00007390.hg.1	Homo sapiens Kruppel-like factor 8	KLF8
548	TC0600008066.hg.1	Homo sapiens kelch domain containing 3	KLHDC3
698	TC0X00010607.hg.1	Homo sapiens kelch-like family member 13	KLHL13
694	TC0X00009256.hg.1	Homo sapiens kelch-like family member 15	KLHL15
418	TC0300009673.hg.1	Homo sapiens kelch-like family member 24	KLHL24
372	TC0200013357.hg.1	Homo sapiens lysine-rich coiled-coil 1	KRCC1
905	TC1700010676.hg.1	Homo sapiens keratin 15, type I	KRT15
907	TC1700010682.hg.1	Homo sapiens keratin 17, type I	KRT17
906	TC1700010677.hg.1	Homo sapiens keratin 19, type I	KRT19
796	TC1200010755.hg.1	Homo sapiens keratin 75, type II	KRT75
701	TC0X00011413.hg.1	Homo sapiens L1 cell adhesion molecule	L1CAM
594	TC0600012885.hg.1	Homo sapiens laminin, alpha 4	LAMA4
378	TC0200016424.hg.1	Homo sapiens limb bud and heart development	LBH
411	TC0300009289.hg.1	Homo sapiens leucine, glutamate and lysine rich 1	LEKR1
292	TC0100012097.hg.1	Homo sapiens lectin, galactoside-binding, soluble, 8	LGALS8
823	TC1300008668.hg.1	Homo sapiens lipoma HMGIC fusion partner	LHFP
734	TC1000012505.hg.1	Homo sapiens phospholysine phosphohistidine inorganic pyrophosphate phosphatase	LHPP
498	TC0500010540.hg.1	Homo sapiens leukemia inhibitory factor receptor alpha	LIFR
497	TC0500010493.hg.1	Homo sapiens LMBR1 domain containing 2	LMBRD2
818	TC1200012778.hg.1	Homo sapiens lamin tail domain containing 1	LMNTD1
304	TC0100014766.hg.1	Homo sapiens lysophosphatidic acid receptor 3	LPAR3
520	TC0600007231.hg.1	Homo sapiens leucine rich repeat containing 16A	LRRC16A
665	TC0800011861.hg.1	Homo sapiens leucine rich repeat containing 6	LRRC6
989	TC2000007991.hg.1	Homo sapiens LSM family member 14B	LSM14B
852	TC1400009698.hg.1	Homo sapiens latent transforming growth factor beta binding protein 2	LTBP2
946	TC1900008113.hg.1	Homo sapiens latent transforming growth factor beta binding protein 4	LTBP4
558	TC0600010688.hg.1	Homo sapiens LYR motif containing 4	LYRM4
353	TC0200010636.hg.1	Homo sapiens microtubule-associated protein 2	MAP2
543	TC0600007825.hg.1	Homo sapiens mitogen-activated protein kinase 14	MAPK14
395	TC0300007432.hg.1	Homo sapiens mitogen-activated protein kinase-activated protein kinase 3	MAPKAPK3
350	TC0200010374.hg.1	Homo sapiens methionyl-tRNA synthetase 2, mitochondrial	MARS2

No.	ID	Category	Symbol
565	TC0600010991.hg.1	Homo sapiens membrane bound O-acyltransferase domain containing 1	MBOAT1
506	TC0500011725.hg.1	Homo sapiens mutated in colorectal cancers	MCC
562	TC0600010867.hg.1	Homo sapiens mitochondrial calcium uniporter regulator 1	MCUR1
579	TC0600011808.hg.1	Homo sapiens mediator complex subunit 20	MED20
504	TC0500011418.hg.1	Homo sapiens myocyte enhancer factor 2C	MEF2C
680	TC0900011379.hg.1	Homo sapiens multiple EGF-like-domains 9	MEGF9
383	TC0200016646.hg.1	Homo sapiens mediator of cell motility 1	MEMO1
777	TC1200007626.hg.1	Homo sapiens methyltransferase like 7A	METTL7A
415	TC0300009602.hg.1	Homo sapiens mitofusin 1	MFN1
919	TC1700012269.hg.1	Homo sapiens MGC57346-CRHR1 readthrough	MGC57346-CRHR1
791	TC1200010022.hg.1	Homo sapiens matrix Gla protein	MGP
531	TC0600007585.hg.1	Homo sapiens MHC class I polypeptide-related sequence B	MICB
999	TC2100007949.hg.1	Homo sapiens MIS18 kinetochore protein A	MIS18A
990	TC2000008379.hg.1	Homo sapiens McKusick-Kaufman syndrome	MKKS
853	TC1400009709.hg.1	Homo sapiens mutL homolog 3	MLH3
879	TC1600010859.hg.1	Homo sapiens mixed lineage kinase domain-like	MLKL
759	TC1100012131.hg.1	Homo sapiens matrix metallopeptidase 1	MMP1
369	TC0200013096.hg.1	Homo sapiens MOB kinase activator 1A	MOB1A
808	TC1200012263.hg.1	Homo sapiens M-phase phosphoprotein 9	MPHOSPH9
283	TC0100010815.hg.1	Homo sapiens major histocompatibility complex, class I-related	MR1
585	TC0600011903.hg.1	Homo sapiens mitochondrial ribosomal protein L14	MRPL14
582	TC0600011859.hg.1	Homo sapiens mitochondrial ribosomal protein L2	MRPL2
434	TC0300012445.hg.1	Homo sapiens mitochondrial ribosomal protein L3	MRPL3
580	TC0600011822.hg.1	Homo sapiens mitochondrial ribosomal protein S10	MRPS10
584	TC0600011884.hg.1	Homo sapiens mitochondrial ribosomal protein S18A	MRPS18A
407	TC0300008964.hg.1	Homo sapiens mitochondrial ribosomal protein S22	MRPS22
654	TC0800010783.hg.1	Homo sapiens musculin	MSC
641	TC0800006692.hg.1	Homo sapiens methionine sulfoxide reductase A	MSRA
703	TC1000007010.hg.1	Homo sapiens methionine sulfoxide reductase B2	MSRB2
380	TC0200016471.hg.1	Homo sapiens MAX dimerization protein 1	MXD1
693	TC0X00008940.hg.1	Homo sapiens matrix-remodelling associated 5	MXRA5
653	TC0800010685.hg.1	Homo sapiens v-myb avian myeloblastosis viral oncogene homolog-like 1	MYBL1
987	TC2000007438.hg.1	Homo sapiens v-myb avian myeloblastosis viral oncogene homolog-like 2	MYBL2

No.	ID	Category	Symbol
954	TC1900009357.hg.1	Homo sapiens myeloid-derived growth factor	MYDGF
766	TC1100013059.hg.1	Homo sapiens myeloma overexpressed	MYEOV
812	TC1200012647.hg.1	Homo sapiens myosin, light chain 6B, alkali, smooth muscle and non-muscle	MYL6B
430	TC0300012212.hg.1	Homo sapiens myosin light chain kinase	MYLK
933	TC1800009290.hg.1	Homo sapiens myosin VB	MYO5B
707	TC1000007847.hg.1	Homo sapiens myopalladin	MYPN
826	TC1300009646.hg.1	Homo sapiens sodium leak channel, non selective	NALCN
895	TC1700009256.hg.1	Homo sapiens nuclear prelamin A recognition factor	NARF
742	TC1100009068.hg.1	Homo sapiens neural cell adhesion molecule 1	NCAM1
443	TC0300013151.hg.1	Homo sapiens neutral cholesterol ester hydrolase 1	NCEH1
922	TC1800006484.hg.1	Homo sapiens NDC80 kinetochore complex component	NDC80
317	TC0100017216.hg.1	Homo sapiens NIMA-related kinase 2	NEK2
800	TC1200010946.hg.1	Homo sapiens nuclear envelope integral membrane protein 1	NEMP1
275	TC0100008517.hg.1	Homo sapiens nuclear factor I/A	NFIA
478	TC0400012792.hg.1	Homo sapiens NIPA-like domain containing 1	NIPAL1
412	TC0300009357.hg.1	Homo sapiens NMD3 ribosome export adaptor	NMD3
358	TC0200011726.hg.1	Homo sapiens nucleolar protein 10	NOL10
967	TC1900011150.hg.1	Homo sapiens nitric oxide synthase interacting protein	NOSIP
851	TC1400009697.hg.1	Homo sapiens Niemann-Pick disease, type C2	NPC2
483	TC0500007077.hg.1	Homo sapiens natriuretic peptide receptor 3	NPR3
872	TC1500009984.hg.1	Homo sapiens neuroplastin	NPTN
332	TC0200007067.hg.1	Homo sapiens nuclear receptor binding protein 1	NRBP1
1004	TC2100008545.hg.1	Homo sapiens nuclear receptor interacting protein 1	NRIP1
750	TC1100010054.hg.1	Homo sapiens nuclear receptor interacting protein 3	NRIP3
748	TC1100009561.hg.1	Homo sapiens neurotrimin	NTM
291	TC0100011999.hg.1	Homo sapiens nucleoside-triphosphatase, cancer-related	NTPCR
867	TC1500006999.hg.1	Homo sapiens nucleolar and spindle associated protein 1	NUSAP1
779	TC1200007810.hg.1	Homo sapiens ORMDL sphingolipid biosynthesis regulator 2	ORMDL2
726	TC1000011382.hg.1	Homo sapiens pantothenate kinase 1	PANK1
668	TC0900008545.hg.1	Homo sapiens pregnancy-associated plasma protein A, pappalysin 1	PAPPA
713	TC1000008354.hg.1	Homo sapiens 3-phosphoadenosine 5-phosphosulfate synthase 2	PAPSS2
869	TC1500007695.hg.1	Homo sapiens progestin and adipoQ receptor family member V	PAQR5
446	TC0300013357.hg.1	Homo sapiens presenilin associated, rhomboid-like	PARL
783	TC1200008642.hg.1	Homo sapiens PARP1 binding protein	PARPBP

No.	ID	Category	Symbol
651	TC0800009970.hg.1	Homo sapiens PDZ binding kinase	PBK
572	TC0600011483.hg.1	Homo sapiens pre-B-cell leukemia homeobox 2	PBX2
405	TC0300008912.hg.1	Homo sapiens propionyl CoA carboxylase, beta polypeptide	PCCB
460	TC0400007169.hg.1	Homo sapiens protocadherin 7	PCDH7
632	TC0700011655.hg.1	Homo sapiens piccolo presynaptic cytomatrix protein	PCLO
775	TC1200007037.hg.1	Homo sapiens phosphodiesterase 3A, cGMP-inhibited	PDE3A
502	TC0500010842.hg.1	Homo sapiens phosphodiesterase 4D, cAMP-specific	PDE4D
556	TC0600009545.hg.1	Homo sapiens phosphodiesterase 7B	PDE7B
643	TC0800006873.hg.1	Homo sapiens platelet-derived growth factor receptor-like	PDGFRL
265	TC0100006931.hg.1	Homo sapiens podoplanin	PDPN
482	TC0500007050.hg.1	Homo sapiens PDZ domain containing 2	PDZD2
832	TC1400007259.hg.1	Homo sapiens pellino E3 ubiquitin protein ligase family member 2	PELI2
262	TC0100006725.hg.1	Homo sapiens period circadian clock 3	PER3
681	TC0900011388.hg.1	Homo sapiens PHD finger protein 19	PHF19
452	TC0300013921.hg.1	Homo sapiens phosphatidylinositol glycan anchor biosynthesis, class X	PIGX
433	TC0300012426.hg.1	Homo sapiens phosphoinositide-3-kinase, regulatory subunit 4	PIK3R4
266	TC0100007207.hg.1	Homo sapiens PTEN induced putative kinase 1	PINK1
893	TC1700008690.hg.1	Homo sapiens phosphatidylinositol transfer protein, cytoplasmic 1	PITPNC1
712	TC1000008054.hg.1	Homo sapiens plasminogen activator, urokinase	PLAU
670	TC0900009461.hg.1	Homo sapiens plasminogen receptor, C-terminal lysine transmembrane protein	PLGRKT
500	TC0500010766.hg.1	Homo sapiens phospholipid phosphatase 1	PLPP1
952	TC1900009076.hg.1	Homo sapiens phospholipid phosphatase 2	PLPP2
325	TC0100018443.hg.1	Homo sapiens phospholipid phosphatase 3	PLPP3
994	TC2000009250.hg.1	Homo sapiens phospholipid transfer protein	PLTP
702	TC1000006937.hg.1	Homo sapiens plexin domain containing 2	PLXDC2
850	TC1400009668.hg.1	Homo sapiens paraneoplastic Ma antigen 1	PNMA1
366	TC0200012652.hg.1	Homo sapiens polyribonucleotide nucleotidyltransferase 1	PNPT1
637	TC0700012636.hg.1	Homo sapiens podocalyxin-like	PODXL
669	TC0900009003.hg.1	Homo sapiens protein-O-mannosyltransferase 1	POMT1
591	TC0600012709.hg.1	Homo sapiens popeye domain containing 3	POPDC3
542	TC0600007792.hg.1	Homo sapiens peroxisome proliferator-activated receptor delta	PPARD
975	TC1900011765.hg.1	Homo sapiens protein phosphatase 1, regulatory subunit 37	PPP1R37
404	TC0300008904.hg.1	Homo sapiens protein phosphatase 2, regulatory subunit B, alpha	PPP2R3A
841	TC1400008333.hg.1	Homo sapiens protein phosphatase 2, regulatory subunit B, gamma	PPP2R5C

No.	ID	Category	Symbol
547	TC0600008064.hg.1	Homo sapiens protein phosphatase 2, regulatory subunit B, delta	PPP2R5D
860	TC1400009982.hg.1	Homo sapiens protein phosphatase 4, regulatory subunit 3A	PPP4R3A
397	TC0300007512.hg.1	Homo sapiens protein kinase C, delta	PRKCD
413	TC0300009468.hg.1	Homo sapiens protein kinase C, iota	PRKCI
985	TC2000007209.hg.1	Homo sapiens protein C receptor, endothelial	PROCR
945	TC1900007882.hg.1	Homo sapiens proline and serine rich 3	PROSER3
532	TC0600007599.hg.1	Homo sapiens proline-rich coiled-coil 2A	PRRC2A
649	TC0800009764.hg.1	Homo sapiens pleckstrin and Sec7 domain containing 3	PSD3
964	TC1900010836.hg.1	Homo sapiens pregnancy specific beta-1-glycoprotein 4	PSG4
963	TC1900010833.hg.1	Homo sapiens pregnancy specific beta-1-glycoprotein 5	PSG5
979	TC1900011967.hg.1	Homo sapiens pregnancy specific beta-1-glycoprotein 9	PSG9
673	TC0900009592.hg.1	Homo sapiens PC4 and SFRS1 interacting protein 1	PSIP1
838	TC1400007947.hg.1	Homo sapiens proteasome 26S subunit, ATPase 1	PSMC1
307	TC0100015192.hg.1	Homo sapiens proline/serine-rich coiled-coil 1	PSRC1
318	TC0100017289.hg.1	Homo sapiens protein tyrosine phosphatase, non-receptor type 14	PTPN14
857	TC1400009922.hg.1	Homo sapiens protein tyrosine phosphatase, non-receptor type 21	PTPN21
801	TC1200011245.hg.1	Homo sapiens protein tyrosine phosphatase, receptor type, B	PTPRB
923	TC1800006589.hg.1	Homo sapiens protein tyrosine phosphatase, receptor type, M	PTPRM
360	TC0200011990.hg.1	Homo sapiens peptidyl-tRNA hydrolase domain containing 1	PTRHD1
984	TC2000007015.hg.1	Homo sapiens phosphorylase, glycogen; brain	PYGB
948	TC1900008343.hg.1	Homo sapiens glutaminyl-peptide cyclotransferase-like	QPCTL
756	TC1100011797.hg.1	Homo sapiens RAB30, member RAS oncogene family	RAB30
988	TC2000007857.hg.1	Homo sapiens ribonucleic acid export 1	RAE1
672	TC0900009479.hg.1	Homo sapiens RAN binding protein 6	RANBP6
561	TC0600010864.hg.1	Homo sapiens RAN binding protein 9	RANBP9
410	TC0300009229.hg.1	Homo sapiens RAP2B, member of RAS oncogene family	RAP2B
834	TC1400007625.hg.1	Homo sapiens RNA binding motif protein 25	RBM25
458	TC0400007120.hg.1	Homo sapiens recombination signal binding protein for immunoglobu-lin kappa J region	RBPJ
646	TC0800007185.hg.1	Homo sapiens RNA binding protein with multiple splicing	RBPMS
269	TC0100007559.hg.1	Homo sapiens regulator of chromosome condensation 1	RCC1
286	TC0100011485.hg.1	Homo sapiens REST corepressor 3	RCOR3
379	TC0200016452.hg.1	Homo sapiens v-rel avian reticuloendotheliosis viral oncogene homolog	REL
817	TC1200012768.hg.1	Homo sapiens RAS-like, estrogen-regulated, growth inhibitor	RERG
731	TC1000012011.hg.1	Homo sapiens regulator of G-protein signaling 10	RGS10

No.	ID	Category	Symbol
809	TC1200012573.hg.1	Homo sapiens RAD9-HUS1-RAD1 interacting nuclear orphan 1	RHNO1
959	TC1900010375.hg.1	Homo sapiens rhophilin, Rho GTPase binding protein 2	RHPN2
536	TC0600007687.hg.1	Homo sapiens ring finger protein 1	RING1
341	TC0200008262.hg.1	Homo sapiens ring finger protein 181	RNF181
515	TC0600007006.hg.1	Homo sapiens ring finger protein 182	RNF182
682	TC0900012225.hg.1	Homo sapiens ring finger protein 38	RNF38
545	TC0600007868.hg.1	Homo sapiens ring finger protein 8, E3 ubiquitin protein ligase	RNF8
787	TC1200009031.hg.1	Homo sapiens ring finger protein, transmembrane 2	RNFT2
424	TC0300011628.hg.1	Homo sapiens roundabout guidance receptor 1	ROBO1
342	TC0200008351.hg.1	Homo sapiens ribose 5-phosphate isomerase A	RPIA
441	TC0300013103.hg.1	Homo sapiens ribosomal protein L22-like 1	RPL22L1
607	TC0600014127.hg.1	Homo sapiens ribosomal protein L7-like 1	RPL7L1
601	TC0600014095.hg.1	Homo sapiens ribonuclease P/MRP 21kDa subunit	RPP21
859	TC1400009973.hg.1	Homo sapiens ribosomal protein S6 kinase, 90kDa, polypeptide 5	RPS6KA5
376	TC0200016402.hg.1	Homo sapiens radical S-adenosyl methionine domain containing 2	RSAD2
723	TC1000010758.hg.1	Homo sapiens rhotekin 2	RTKN2
848	TC1400009329.hg.1	Homo sapiens reticulon 1	RTN1
453	TC0300014041.hg.1	Homo sapiens RuvB-like AAA ATPase 1	RUVBL1
609	TC0600014191.hg.1	Homo sapiens sterile alpha motif domain containing 5	SAMD5
466	TC0400009330.hg.1	Homo sapiens Sin3A-associated protein, 30kDa	SAP30
469	TC0400011180.hg.1	Homo sapiens stearoyl-CoA desaturase 5	SCD5
359	TC0200011898.hg.1	Homo sapiens syndecan 1	SDC1
914	TC1700012110.hg.1	Homo sapiens secreted and transmembrane 1	SECTM1
699	TC0X00010643.hg.1	Homo sapiens septin 6	SEPT6
270	TC0100007638.hg.1	Homo sapiens serine incorporator 2	SERINC2
285	TC0100011466.hg.1	Homo sapiens SERTA domain containing 4	SERTAD4
592	TC0600012801.hg.1	Homo sapiens sestrin 1	SESN1
758	TC1100012019.hg.1	Homo sapiens sestrin 3	SESN3
595	TC0600013433.hg.1	Homo sapiens splicing factor 3b, subunit 5, 10kDa	SF3B5
652	TC0800010234.hg.1	Homo sapiens secreted frizzled-related protein 1	SFRP1
387	TC0200016687.hg.1	Homo sapiens sideroflexin 5	SFXN5
686	TC0X00007744.hg.1	Homo sapiens SH3 domain binding glutamate-rich protein like	SH3BGRL
476	TC0400012378.hg.1	Homo sapiens SH3 domain containing ring finger 1	SH3RF1
461	TC0400007371.hg.1	Homo sapiens shisa family member 3	SHISA3

No.	ID	Category	Symbol
322	TC0100017748.hg.1	Homo sapiens signal-induced proliferation-associated 1 like 2	SIPA1L2
849	TC1400009353.hg.1	Homo sapiens SIX homeobox 4	SIX4
309	TC0100015234.hg.1	Homo sapiens solute carrier family 16, member 4	SLC16A4
722	TC1000010722.hg.1	Homo sapiens solute carrier family 16, member 9	SLC16A9
939	TC1900007419.hg.1	Homo sapiens solute carrier family 25, member 42	SLC25A42
862	TC1400010616.hg.1	Homo sapiens solute carrier family 38, member 6	SLC38A6
435	TC0300012484.hg.1	Homo sapiens solute carrier organic anion transporter family, member 2A1	SLCO2A1
509	TC0500012760.hg.1	Homo sapiens slit guidance ligand 3	SLIT3
700	TC0X00010785.hg.1	Homo sapiens SWI/SNF related, matrix associated, actin dependent regulator of chromatin, subfamily a, member 1	SMARCA1
797	TC1200010900.hg.1	Homo sapiens SWI/SNF related, matrix associated, actin dependent regulator of chromatin, subfamily c, member 2	SMARCC2
382	TC0200016637.hg.1	Homo sapiens structural maintenance of chromosomes 6	SMC6
912	TC1700011451.hg.1	Homo sapiens SMAD specific E3 ubiquitin protein ligase 2	SMURF2
538	TC0600007769.hg.1	Homo sapiens small nuclear ribonucleoprotein polypeptide C	SNRPC
728	TC1000011487.hg.1	Homo sapiens sorbin and SH3 domain containing 1	SORBS1
477	TC0400012644.hg.1	Homo sapiens sorbin and SH3 domain containing 2	SORBS2
308	TC0100015194.hg.1	Homo sapiens sortilin 1	SORT1
745	TC1100009397.hg.1	Homo sapiens sperm autoantigenic protein 17	SPA17
836	TC1400007899.hg.1	Homo sapiens spermatogenesis associated 7	SPATA7
508	TC0500012312.hg.1	Homo sapiens sprouty RTK signaling antagonist 4	SPRY4
263	TC0100006773.hg.1	Homo sapiens splA/ryanodine receptor domain and SOCS box containing 1	SPSB1
855	TC1400009787.hg.1	Homo sapiens serine palmitoyltransferase, long chain base subunit 2	SPTLC2
983	TC2000006736.hg.1	Homo sapiens serine palmitoyltransferase, long chain base subunit 3	SPTLC3
687	TC0X00007919.hg.1	Homo sapiens sushi-repeat containing protein, X-linked 2	SRPX2
394	TC0300007164.hg.1	Homo sapiens synovial sarcoma translocation gene on chromosome 18-like 2	SS18L2
371	TC0200013298.hg.1	Homo sapiens ST3 beta-galactoside alpha-2,3-sialyltransferase 5	ST3GAL5
437	TC0300012534.hg.1	Homo sapiens stromal antigen 1	STAG1
714	TC1000008385.hg.1	Homo sapiens STAM binding protein-like 1	STAMBPL1
650	TC0800009891.hg.1	Homo sapiens stanniocalcin 1	STC1
625	TC0700010443.hg.1	Homo sapiens STEAP family member 1B	STEAP1B
619	TC0700008293.hg.1	Homo sapiens STEAP family member 2, metalloreductase	STEAP2
689	TC0X00008437.hg.1	Homo sapiens serine/threonine protein kinase 26	STK26
577	TC0600011661.hg.1	Homo sapiens serine/threonine kinase 38	STK38

No.	ID	Category	Symbol
300	TC0100013369.hg.1	Homo sapiens stathmin 1	STMN1
370	TC0200013232.hg.1	Homo sapiens succinate-CoA ligase, alpha subunit	SUCLG1
1003	TC2100008385.hg.1	Homo sapiens small ubiquitin-like modifier 3	SUMO3
1017	TC2200009353.hg.1	Homo sapiens Sad1 and UNC84 domain containing 2	SUN2
421	TC0300010668.hg.1	Homo sapiens sushi domain containing 5	SUSD5
604	TC0600014111.hg.1	Homo sapiens synaptic Ras GTPase activating protein 1	SYNGAP1
280	TC0100010140.hg.1	Homo sapiens synaptotagmin XI	SYT11
743	TC1100009169.hg.1	Homo sapiens transgelin	TAGLN
810	TC1200012584.hg.1	Homo sapiens TAP binding protein-like	TAPBPL
459	TC0400007128.hg.1	Homo sapiens TBC1 domain family, member 19	TBC1D19
581	TC0600011843.hg.1	Homo sapiens tubulin folding cofactor C	TBCC
530	TC0600007565.hg.1	Homo sapiens transcription factor 19	TCF19
786	TC1200008726.hg.1	Homo sapiens t-complex 11, testis-specific-like 2	TCP11L2
566	TC0600011067.hg.1	Homo sapiens tyrosyl-DNA phosphodiesterase 2	TDP2
493	TC0500009342.hg.1	Homo sapiens teneurin transmembrane protein 2	TENM2
613	TC0600014362.hg.1	Homo sapiens transcription factor B1, mitochondrial	TFB1M
667	TC0900008202.hg.1	Homo sapiens transforming growth factor, beta receptor 1	TGFBR1
998	TC2000009914.hg.1	Homo sapiens TGFB-induced factor homeobox 2	TGIF2
993	TC2000009058.hg.1	Homo sapiens transglutaminase 2	TGM2
1008	TC2200008067.hg.1	Homo sapiens THAP domain containing 7	THAP7
904	TC1700010230.hg.1	Homo sapiens TGFB1-induced anti-apoptotic factor 1	TIAF1
799	TC1200010917.hg.1	Homo sapiens timeless circadian clock	TIMELESS
902	TC1700010209.hg.1	Homo sapiens TLC domain containing 1	TLCD1
303	TC0100014392.hg.1	Homo sapiens TM2 domain containing 1	TM2D1
440	TC0300012773.hg.1	Homo sapiens transmembrane 4 L six family member 1	TM4SF1
776	TC1200007499.hg.1	Homo sapiens transmembrane protein 106C	TMEM106C
598	TC0600014069.hg.1	Homo sapiens transmembrane protein 14B	TMEM14B
597	TC0600014068.hg.1	Homo sapiens transmembrane protein 14C	TMEM14C
474	TC0400012150.hg.1	Homo sapiens transmembrane protein 154	TMEM154
813	TC1200012666.hg.1	Homo sapiens transmembrane protein 19	TMEM19
674	TC0900010386.hg.1	Homo sapiens transmembrane protein 2	TMEM2
457	TC0300014081.hg.1	Homo sapiens transmembrane protein 41A	TMEM41A
1000	TC2100007996.hg.1	Homo sapiens transmembrane protein 50B	TMEM50B
658	TC0800011075.hg.1	Homo sapiens transmembrane protein 55A	TMEM55A

No.	ID	Category	Symbol
320	TC0100017500.hg.1	Homo sapiens transmembrane protein 63A	TMEM63A
972	TC1900011743.hg.1	Homo sapiens transmembrane protein 91	TMEM91
884	TC1700007360.hg.1	Homo sapiens transmembrane protein 97	TMEM97
917	TC1700012231.hg.1	Homo sapiens transmembrane protein 98	TMEM98
679	TC0900011305.hg.1	Homo sapiens tenascin C	TNC
662	TC0800011611.hg.1	Homo sapiens tumor necrosis factor receptor superfamily, member 11b	TNFRSF11B
586	TC0600011960.hg.1	Homo sapiens tumor necrosis factor receptor superfamily, member 21	TNFRSF21
909	TC1700011121.hg.1	Homo sapiens transducer of ERBB2, 1	TOB1
899	TC1700009651.hg.1	Homo sapiens tumor protein p53	TP53
753	TC1100010639.hg.1	Homo sapiens tumor protein p53 inducible protein 11	TP53I11
553	TC0600009353.hg.1	Homo sapiens tumor protein D52-like 1	TPD52L1
587	TC0600012040.hg.1	Homo sapiens translocation associated membrane protein 2	TRAM2
807	TC1200012161.hg.1	Homo sapiens TP53 regulated inhibitor of apoptosis 1	TRIAP1
764	TC1100012957.hg.1	Homo sapiens tripartite motif containing 22	TRIM22
456	TC0300014075.hg.1	Homo sapiens tripartite motif containing 59	TRIM59
676	TC0900010958.hg.1	Homo sapiens tRNA methyltransferase O	TRMO
399	TC0300008171.hg.1	Homo sapiens tRNA methyltransferase 10C, mitochondrial RNase P subunit	TRMT10C
450	TC0300013888.hg.1	Homo sapiens TSC22 domain family, member 2	TSC22D2
951	TC1900008839.hg.1	Homo sapiens TSEN34 tRNA splicing endonuclease subunit	TSEN34
636	TC0700012416.hg.1	Homo sapiens tetraspanin 12	TSPAN12
356	TC0200011548.hg.1	Homo sapiens tumor suppressing subtransferable candidate 1	TSSC1
858	TC1400009967.hg.1	Homo sapiens tetratricopeptide repeat domain 7B	TTC7B
279	TC0100009561.hg.1	Homo sapiens transcription termination factor, RNA polymerase II	TTF2
921	TC1800006448.hg.1	Homo sapiens thymidylate synthetase	TYMS
449	TC0300013881.hg.1	Homo sapiens U2 snRNP-associated SURP domain containing	U2SURP
744	TC1100009330.hg.1	Homo sapiens ubiquitin associated and SH3 domain containing B	UBASH3B
391	TC0300006840.hg.1	Homo sapiens ubiquitin-conjugating enzyme E2E 1	UBE2E1
390	TC0300006827.hg.1	Homo sapiens ubiquitin-conjugating enzyme E2E 2	UBE2E2
970	TC1900011470.hg.1	Homo sapiens ubiquitin-conjugating enzyme E2S	UBE2S
315	TC0100016952.hg.1	Homo sapiens ubiquitin-conjugating enzyme E2T	UBE2T
354	TC0200011138.hg.1	Homo sapiens UDP glucuronosyltransferase 1 family, polypeptide A1	UGT1A1
539	TC0600007771.hg.1	Homo sapiens UHRF1 binding protein 1	UHRF1BP1
596	TC0600013542.hg.1	Homo sapiens UL16 binding protein 3	ULBP3
402	TC0300008604.hg.1	Homo sapiens uridine monophosphate synthetase	UMPS

No.	ID	Category	Symbol
958	TC1900010286.hg.1	Homo sapiens ubiquinol-cytochrome c reductase, Rieske iron-sulfur polypeptide 1	UQCRFS1
724	TC1000011026.hg.1	Homo sapiens ubiquitin specific peptidase 54	USP54
425	TC0300011706.hg.1	Homo sapiens vestigial-like family member 3	VGLL3
329	TC0200006831.hg.1	Homo sapiens visinin-like 1	VSNL1
688	TC0X00008001.hg.1	Homo sapiens WW domain binding protein 5	WBP5
333	TC0200007111.hg.1	Homo sapiens WD repeat domain 43	WDR43
574	TC0600011534.hg.1	Homo sapiens WD repeat domain 46	WDR46
338	TC0200008080.hg.1	Homo sapiens WD repeat domain 54	WDR54
386	TC0200016681.hg.1	Homo sapiens WD repeat domain 92	WDR92
267	TC0100007505.hg.1	Homo sapiens WD and tetratricopeptide repeats 1	WDTC1
770	TC1200006469.hg.1	Homo sapiens wingless-type MMTV integration site family, member 5B	WNT5B
883	TC1700007319.hg.1	Homo sapiens WD repeat and SOCS box containing 1	WSB1
583	TC0600011878.hg.1	Homo sapiens exportin 5	XPO5
980	TC1900011968.hg.1	Homo sapiens X-ray repair complementing defective repair in Chinese hamster cells 1	XRCC1
780	TC1200007906.hg.1	Homo sapiens XRCC6 binding protein 1	XRCC6BP1
875	TC1600009580.hg.1	Homo sapiens xylosyltransferase I	XYLT1
781	TC1200008134.hg.1	Homo sapiens YEATS domain containing 4	YEATS4
930	TC1800009056.hg.1	Homo sapiens zinc binding alcohol dehydrogenase domain containing 2	ZADH2
605	TC0600014112.hg.1	Homo sapiens zinc finger and BTB domain containing 9	ZBTB9
837	TC1400007906.hg.1	Homo sapiens zinc finger CCCH-type containing 14	ZC3H14
696	TC0X00009890.hg.1	Homo sapiens zinc finger, C4H2 domain containing	ZC4H2
444	TC0300013255.hg.1	Homo sapiens zinc finger, matrin-type 3	ZMAT3
569	TC0600011197.hg.1	Homo sapiens zinc finger protein 184	ZNF184
612	TC0600014294.hg.1	Homo sapiens zinc finger protein 318	ZNF318
969	TC1900011329.hg.1	Homo sapiens zinc finger protein 415	ZNF415
934	TC1900006932.hg.1	Homo sapiens ZNF559-ZNF177 readthrough	ZNF559-ZNF177
947	TC1900008172.hg.1	Homo sapiens zinc finger protein 574	ZNF574
507	TC0500011896.hg.1	Homo sapiens zinc finger protein 608	ZNF608
414	TC0300009600.hg.1	Homo sapiens zinc finger protein 639	ZNF639
815	TC1200012723.hg.1	Homo sapiens zinc finger protein 664	ZNF664
656	TC0800010918.hg.1	Homo sapiens zinc finger protein 704	ZNF704
541	TC0600007789.hg.1	Homo sapiens zinc finger protein 76	ZNF76
982	TC1900012057.hg.1	Homo sapiens zinc finger protein 814	ZNF814

No.	ID	Category	Symbol
940	TC1900007502.hg.1	Homo sapiens zinc finger protein 85	ZNF85
887	TC1700007634.hg.1	Homo sapiens zinc finger, HIT-type containing 3	ZNHIT3
599	TC0600014093.hg.1	Homo sapiens zinc ribbon domain containing 1	ZNRD1
981	TC1900012053.hg.1	Homo sapiens zinc finger and SCAN domain containing 5A	ZSCAN5A
711	TC1000008046.hg.1	Homo sapiens fucosyltransferase 11 (alpha (1,3) fucosyltransferase)	FUT11
677	TC0900011072.hg.1	Homo sapiens ATP-binding cassette, sub-family A (ABC1), member 1	ABCA1
913	TC1700011574.hg.1	Homo sapiens ATP-binding cassette, sub-family A (ABC1), member 8	ABCA8
623	TC0700009690.hg.1	Homo sapiens ATP-binding cassette, sub-family B (MDR/TAP), member 8	ABCB8
447	TC0300013360.hg.1	Homo sapiens ATP-binding cassette, sub-family C (CFTR/MRP), member 5	ABCC5
557	TC0600009862.hg.1	Homo sapiens A kinase (PRKA) anchor protein 12	AKAP12
727	TC1000011400.hg.1	Homo sapiens ankyrin repeat domain 1 (cardiac muscle)	ANKRD1
468	TC0400010519.hg.1	Homo sapiens amyloid beta (A4) precursor protein-binding, family B, member 2	APBB2
622	TC0700009536.hg.1	Homo sapiens Rho guanine nucleotide exchange factor (GEF) 5	ARHGEF5
949	TC1900008551.hg.1	Homo sapiens BCL2-like 12 (proline rich)	BCL2L12
517	TC0600007087.hg.1	Homo sapiens CAP, adenylate cyclase-associated protein, 2 (yeast)	CAP2
886	TC1700007557.hg.1	Homo sapiens chemokine (C-C motif) ligand 2	CCL2
385	TC0200016648.hg.1	Homo sapiens CDC42 effector protein (Rho GTPase binding) 3	CDC42EP3
496	TC0500010282.hg.1	Homo sapiens cadherin 12, type 2 (N-cadherin 2)	CDH12
544	TC0600007847.hg.1	Homo sapiens cyclin-dependent kinase inhibitor 1A (p21, Cip1)	CDKN1A
774	TC1200006888.hg.1	Homo sapiens cyclin-dependent kinase inhibitor 1B (p27, Kip1)	CDKN1B
274	TC0100008260.hg.1	Homo sapiens cyclin-dependent kinase inhibitor 2C (p18, inhibits CDK4)	CDKN2C
962	TC1900010807.hg.1	Homo sapiens carcinoembryonic antigen-related cell adhesion molecule 1 (biliary glycoprotein)	CEACAM1
843	TC1400008919.hg.1	Homo sapiens cofilin 2 (muscle)	CFL2
589	TC0600012521.hg.1	Homo sapiens cannabinoid receptor 1 (brain)	CNR1
741	TC1100008469.hg.1	Homo sapiens serpin peptidase inhibitor, clade H (heat shock protein 47), member 1, (collagen binding protein 1)	SERPINH1
328	TC0200006788.hg.1	Homo sapiens DEAD (Asp-Glu-Ala-Asp) box helicase 1	DDX1
888	TC1700007641.hg.1	Homo sapiens dehydrogenase/reductase (SDR family) member 11	DHRS11
903	TC1700010221.hg.1	Homo sapiens dehydrogenase/reductase (SDR family) member 13	DHRS13
898	TC1700009619.hg.1	Homo sapiens discs, large homolog 4 (Drosophila)	DLG4
847	TC1400009248.hg.1	Homo sapiens discs, large (Drosophila) homolog-associated protein 5	DLGAP5
835	TC1400007691.hg.1	Homo sapiens dihydrolipoamide S-succinyltransferase (E2 component of 2-oxo-glutarate complex)	DLST

No.	ID	Category	Symbol
488	TC0500008054.hg.1	Homo sapiens polymerase (RNA) III (DNA directed) polypeptide G (32kD)	POLR3G
419	TC0300009706.hg.1	Homo sapiens polymerase (RNA) II (DNA directed) polypeptide H	POLR2H
451	TC0300013911.hg.1	Homo sapiens DnaJ (Hsp40) homolog, subfamily B, member 11	DNAJB11
445	TC0300013296.hg.1	Homo sapiens DnaJ (Hsp40) homolog, subfamily C, member 19	DNAJC19
772	TC1200006653.hg.1	Homo sapiens enolase 2 (gamma, neuronal)	ENO2
290	TC0100011770.hg.1	Homo sapiens epoxide hydrolase 1, microsomal (xenobiotic)	EPHX1
638	TC0700012959.hg.1	Homo sapiens enhancer of zeste homolog 2 (Drosophila)	EZH2
503	TC0500011194.hg.1	Homo sapiens coagulation factor II (thrombin) receptor-like 2	F2RL2
870	TC1500008029.hg.1	Homo sapiens fumarylacetoacetate hydrolase (fumarylacetoacetase)	FAH
749	TC1100009969.hg.1	Homo sapiens amyloid beta (A4) precursor protein-binding, family B, member 1 (Fe65)	APBB1
773	TC1200006787.hg.1	Homo sapiens GABA(A) receptor-associated protein like 1	GABARAPL1
335	TC0200007297.hg.1	Homo sapiens gem (nuclear organelle) associated protein 6	GEMIN6
974	TC1900011764.hg.1	Homo sapiens gem (nuclear organelle) associated protein 7	GEMIN7
830	TC1400007154.hg.1	Homo sapiens guanine nucleotide binding protein (G protein), gamma 2	GNG2
1005	TC2200006614.hg.1	Homo sapiens glycoprotein Ib (platelet), beta polypeptide	GP1BB
344	TC0200009189.hg.1	Homo sapiens glycophorin C (Gerbich blood group)	GYPC
966	TC1900011113.hg.1	Homo sapiens glycogen synthase 1 (muscle)	GYS1
403	TC0300008768.hg.1	Homo sapiens 5-hydroxymethylcytosine (hmC) binding, ES cell-specific	HMCES
281	TC0100010389.hg.1	Homo sapiens hydroxysteroid (17-beta) dehydrogenase 7	HSD17B7
298	TC0100013272.hg.1	Homo sapiens 5-hydroxytryptamine (serotonin) receptor 1D, G protein-coupled	HTR1D
588	TC0600012061.hg.1	Homo sapiens intestinal cell (MAK-like) kinase	ICK
821	TC1300007870.hg.1	Homo sapiens integrin, beta-like 1 (with EGF-like repeat domains)	ITGBL1
760	TC1100012229.hg.1	Homo sapiens KDEL (Lys-Asp-Glu-Leu) containing 2	KDELC2
819	TC1300007491.hg.1	Homo sapiens Kruppel-like factor 5 (intestinal)	KLF5
944	TC1900007878.hg.1	Homo sapiens lysine (K)-specific methyltransferase 2B	KMT2B
825	TC1300008840.hg.1	Homo sapiens lymphocyte cytosolic protein 1 (L-plastin)	LCP1
882	TC1700007105.hg.1	Homo sapiens lethal giant larvae homolog 1 (Drosophila)	LLGL1
296	TC0100012889.hg.1	Homo sapiens MAD2 mitotic arrest deficient-like 2 (yeast)	MAD2L2
757	TC1100011857.hg.1	Homo sapiens malic enzyme 3, NADP(+)-dependent, mitochondrial	ME3
657	TC0800011041.hg.1	Homo sapiens matrix metallopeptidase 16 (membrane-inserted)	MMP16
889	TC1700007779.hg.1	Homo sapiens male-specific lethal 1 homolog (Drosophila)	MSL1
436	TC0300012527.hg.1	Homo sapiens male-specific lethal 2 homolog (Drosophila)	MSL2

No.	ID	Category	Symbol
334	TC0200007257.hg.1	Homo sapiens NADH dehydrogenase (ubiquinone) complex I, assembly factor 7	NDUFAF7
845	TC1400009141.hg.1	Homo sapiens ninein (GSK3B interacting protein)	NIN
941	TC1900007754.hg.1	Homo sapiens nudix (nucleoside diphosphate linked moiety X)-type motif 19	NUDT19
396	TC0300007454.hg.1	Homo sapiens poly (ADP-ribose) polymerase family, member 3	PARP3
861	TC1400010612.hg.1	Homo sapiens pecanex-like 4 (Drosophila)	PCNXL4
486	TC0500007365.hg.1	Homo sapiens pelota homolog (Drosophila)	PELO
302	TC0100014040.hg.1	Homo sapiens phosphoinositide-3-kinase, regulatory subunit 3 (gamma)	PIK3R3
997	TC2000009887.hg.1	Homo sapiens phospholipase C, beta 1 (phosphoinositide-specific)	PLCB1
844	TC1400009108.hg.1	Homo sapiens polymerase (DNA directed), epsilon 2, accessory subunit	POLE2
340	TC0200008103.hg.1	Homo sapiens polymerase (DNA-directed), epsilon 4, accessory subunit	POLE4
911	TC1700011448.hg.1	Homo sapiens polymerase (DNA directed), gamma 2, accessory subunit	POLG2
550	TC0600008099.hg.1	Homo sapiens polymerase (DNA directed), eta	POLH
428	TC0300012164.hg.1	Homo sapiens polymerase (DNA directed), theta	POLQ
388	TC0200016702.hg.1	Homo sapiens polymerase (RNA) I polypeptide A, 194kDa	POLR1A
549	TC0600008095.hg.1	Homo sapiens polymerase (RNA) I polypeptide C, 30kDa	POLR1C
683	TC0X00007190.hg.1	Homo sapiens porcupine homolog (Drosophila)	PORCN
704	TC1000007461.hg.1	Homo sapiens Ras association (RalGDS/AF-6) domain family member 4	RASSF4
294	TC0100012278.hg.1	Homo sapiens saccharopine dehydrogenase (putative)	SCCPDH
856	TC1400009842.hg.1	Homo sapiens sel-1 suppressor of lin-12-like (C. elegans)	SEL1L
640	TC0700013578.hg.1	Homo sapiens sema domain, immunoglobulin domain (Ig), short basic domain, secreted, (semaphorin) 3A	SEMA3A
633	TC0700011675.hg.1	Homo sapiens sema domain, immunoglobulin domain (Ig), short basic domain, secreted, (semaphorin) 3D	SEMA3D
610	TC0600014217.hg.1	Homo sapiens serpin peptidase inhibitor, clade B (ovalbumin), member 1	SERPINB1
932	TC1800009242.hg.1	Homo sapiens serpin peptidase inhibitor, clade B (ovalbumin), member 2	SERPINB2
490	TC0500008632.hg.1	Homo sapiens solute carrier family 22 (organic cation/carnitine transporter), member 5	SLC22A5
920	TC1700012315.hg.1	Homo sapiens solute carrier family 25 (mitochondrial carrier; dicarboxylate transporter), member 10	SLC25A10
423	TC0300011038.hg.1	Homo sapiens solute carrier family 25 (carnitine/acylcarnitine translocase), member 20	SLC25A20
408	TC0300008989.hg.1	Homo sapiens solute carrier family 25 (pyrimidine nucleotide carrier), member 36	SLC25A36
467	TC0400009518.hg.1	Homo sapiens solute carrier family 25 (mitochondrial carrier; adenine nucleotide translocator), member 4	SLC25A4

No.	ID	Category	Symbol
491	TC0500009076.hg.1	Homo sapiens solute carrier family 26 (anion exchanger), member 2	SLC26A2
708	TC1000007954.hg.1	Homo sapiens solute carrier family 29 (equilibrative nucleoside transporter), member 3	SLC29A3
1013	TC2200009230.hg.1	Homo sapiens solute carrier family 2 (facilitated glucose transporter), member 11	SLC2A11
794	TC1200010415.hg.1	Homo sapiens solute carrier family 2 (facilitated glucose transporter), member 13	SLC2A13
349	TC0200010328.hg.1	Homo sapiens solute carrier family 39 (zinc transporter), member 10	SLC39A10
481	TC0400012949.hg.1	Homo sapiens solute carrier family 39 (zinc transporter), member 8	SLC39A8
362	TC0200012066.hg.1	Homo sapiens solute carrier family 5 (sodium/multivitamin and iodide cotransporter), member 6	SLC5A6
692	TC0X00008794.hg.1	Homo sapiens solute carrier family 6 (neurotransmitter transporter), member 8	SLC6A8
642	TC0800006869.hg.1	Homo sapiens solute carrier family 7 (cationic amino acid transporter, y+ system), member 2	SLC7A2
276	TC0100008787.hg.1	Homo sapiens ST6 (alpha-N-acetyl-neuraminyl-2,3-beta-galactosyl-1, 3)-N-acetylgalactosaminide alpha-2,6-sialyltransferase 5	ST6GALNAC5
327	TC0200006665.hg.1	Homo sapiens TATA box binding protein (TBP)-associated factor, RNA polymerase I, B, 63kDa	TAF1B
719	TC1000008769.hg.1	Homo sapiens TAF5 RNA polymerase II, TATA box binding protein (TBP)-associated factor, 100kDa	TAF5
576	TC0600011536.hg.1	Homo sapiens TAP binding protein (tapasin)	TAPBP
473	TC0400011978.hg.1	Homo sapiens TBC1 domain family, member 9 (with GRAM domain)	TBC1D9
559	TC0600010777.hg.1	Homo sapiens transcription factor AP-2 alpha (activating enhancer binding protein 2 alpha)	TFAP2A
438	TC0300012654.hg.1	Homo sapiens transcription factor Dp-2 (E2F dimerization partner 2)	TFDP2
392	TC0300006925.hg.1	Homo sapiens transforming growth factor, beta receptor II (70/80kDa)	TGFBR2
442	TC0300013146.hg.1	Homo sapiens tumor necrosis factor (ligand) superfamily, member 10	TNFSF10
795	TC1200010559.hg.1	Homo sapiens vitamin D (1,25- dihydroxyvitamin D3) receptor	VDR
573	TC0600011531.hg.1	Homo sapiens vacuolar protein sorting 52 homolog (S. cerevisiae)	VPS52
1009	TC2200008099.hg.1	Homo sapiens YdjC homolog (bacterial)	YDJC
525	TC0600007313.hg.1	Homo sapiens activator of basal transcription 1 (ABT1), mRNA.	ABT1
1014	TC2200009270.hg.1	Homo sapiens apolipoprotein B mRNA editing enzyme, catalytic polypeptide-like 3B (APOBEC3B), transcript variant 2, mRNA.	APOBEC3B
1015	TC2200009271.hg.1	Homo sapiens apolipoprotein B mRNA editing enzyme, catalytic polypeptide-like 3C (APOBEC3C), mRNA.	APOBEC3C
1016	TC2200009273.hg.1	Homo sapiens apolipoprotein B mRNA editing enzyme, catalytic polypeptide-like 3F (APOBEC3F), transcript variant 2, mRNA.	APOBEC3F
788	TC1200009590.hg.1	Homo sapiens decapping mRNA 1B (DCP1B), mRNA.	DCP1B
710	TC1000007990.hg.1	Homo sapiens DNA-damage-inducible transcript 4 (DDIT4), mRNA.	DDIT4
363	TC0200012073.hg.1	Homo sapiens general transcription factor IIIC, polypeptide 2, beta	GTF3C2

No.	ID	Category	Symbol
		110kDa (GTF3C2), transcript variant 2, mRNA.	
400	TC0300008518.hg.1	Homo sapiens general transcription factor IIE, polypeptide 1, alpha 56kDa (GTF2E1), mRNA.	GTF2E1
603	TC0600014099.hg.1	Homo sapiens general transcription factor IIH, polypeptide 4, 52kDa (GTF2H4), mRNA.	GTF2H4
439	TC0300012764.hg.1	Homo sapiens helicase-like transcription factor (HLTF), transcript variant 1, mRNA.	HLTF
554	TC0600009362.hg.1	Homo sapiens hes-related family bHLH transcription factor with YRPW motif 2 (HEY2), mRNA.	HEY2
655	TC0800010894.hg.1	Homo sapiens hes-related family bHLH transcription factor with YRPW motif 1 (HEY1), transcript variant 2, mRNA.	HEY1
448	TC0300013420.hg.1	Homo sapiens insulin-like growth factor 2 mRNA binding protein 2 (IGF2BP2), transcript variant 2, mRNA.	IGF2BP2
606	TC0600014123.hg.1	Homo sapiens nuclear transcription factor Y, alpha (NFYA), transcript variant 1, mRNA.	NFYA
876	TC1600009865.hg.1	Homo sapiens nuclear protein, transcriptional regulator, 1 (NUPR1), transcript variant 1, mRNA.	NUPR1
310	TC0100015330.hg.1	Homo sapiens putative homeodomain transcription factor 1 (PHTF1), mRNA.	PHTF1
618	TC0700008149.hg.1	Homo sapiens putative homeodomain transcription factor 2 (PHTF2), transcript variant 1, mRNA.	PHTF2
798	TC1200010908.hg.1	Homo sapiens signal transducer and activator of transcription 2, 113kDa (STAT2), transcript variant 1, mRNA.	STAT2
600	TC0600014094.hg.1	Homo sapiens tripartite motif-containing 39, mRNA (cDNA clone MGC:2475 IMAGE:3051389), complete cds.	TRIM39
784	TC1200008667.hg.1	Transcript Identified by AceView, Entrez Gene ID(s) 7184	HSP90B1
915	TC1700012177.hg.1	WSC domain containing 1[gene_biotype:protein_coding transcript_biotype:processed_transcript]	WSCD1
971	TC1900011730.hg.1	translocase of inner mitochondrial membrane 50 homolog (S. cerevisiae) [gene_biotype:protein_coding transcript_biotype:retained_intron]	TIMM50
345	TC0200009393.hg.1	Homo sapiens uncharacterized LOC101928161 (LOC101928161), long non-coding RNA.	LOC101928161
389	TC0200016770.hg.1	Homo sapiens microRNA 6809 (MIR6809), microRNA.	MIR6809
1007	TC2200008036.hg.1	ubiquitin specific peptidase 41[gene_biotype:protein_coding transcript_biotype:protein_coding]	CAOG_005894
512	TC0500013205.hg.1	calpastatin [gene_biotype:protein_coding transcript_biotype:nonsense_mediated_decay]	CAST
521	TC0600007266.hg.1	Memczak2013 ANTISENSE, CDS, coding, INTERNAL best transcript NM_005319	HIST1H1C
527	TC0600007535.hg.1	ATP-binding cassette, sub-family F (GCN20), member 1 [gene_biotype:protein_coding transcript_biotype:protein_coding]	ABCF1
528	TC0600007540.hg.1	chromosome 6 open reading frame 136 [gene_biotype:protein_coding transcript_biotype:protein_coding]	C6orf136
534	TC0600007616.hg.1	heat shock 70kDa protein 1B [gene_biotype:protein_coding transcript_biotype:protein_coding]	HSPA1B

No.	ID	Category	Symbol
575	TC0600011535.hg.1	ral guanine nucleotide dissociation stimulator-like 2 [gene_biotype:protein_coding transcript_biotype:protein_coding]	RGL2
608	TC0600014140.hg.1	Salzman2013 ANNOTATED, CDS, coding, OVCODE, OVEXON, UTR3 best transcript NM_000947	PRIM2
661	TC0800011566.hg.1	Transcript Identified by AceView, Entrez Gene ID(s) 5885	RAD21
1018	TSUnmapped00000211.hg.1	SERTA domain containing 4 [gene_biotype:protein_coding tran-script_biotype:processed_transcript]	SERTAD4
1019	TSUnmapped00000269.hg.1	SERTA domain containing 4 [gene_biotype:protein_coding tran-script_biotype:processed_transcript]	SERTAD4
1020	TSUnmapped00000328.hg.1	diacylglycerol kinase, delta 130kDa [gene_biotype:protein_coding transcript_biotype:retained_intron]	DGKD
1021	TSUnmapped00000344.hg.1	fibrillarin [gene_biotype:protein_coding tran-script_biotype:nonsense_mediated_decay]	FBL
1022	TSUnmapped00000398.hg.1	K(lysine) acetyltransferase 6B [gene_biotype:protein_coding tran-script_biotype:protein_coding]	KAT6B
1023	TSUnmapped00000445.hg.1	fibrillarin [gene_biotype:protein_coding tran-script_biotype:protein_coding]	FBL
1024	TSUnmapped00000538.hg.1	diacylglycerol kinase, delta 130kDa [gene_biotype:protein_coding transcript_biotype:retained_intron]	DGKD
1025	TSUnmapped00000585.hg.1	proj_havana:known chromo-some:GRCh38:CHR_HG2235_PATCH:66410247:66417728:-1 gene:ENSG00000282243 gene_biotype:protein_coding tran-script_biotype:processed_transcript	ENSG00000282243
1026	TSUnmapped00000639.hg.1	SERTA domain containing 4 [gene_biotype:protein_coding tran-script_biotype:protein_coding]	SERTAD4
1027	TSUnmapped00000732.hg.1	Glucose-6-phosphate translocase [gene_biotype:protein_coding tran-script_biotype:protein_coding]	SLC37A4
1028	TSUnmapped00000776.hg.1	fibrillarin [gene_biotype:protein_coding tran-script_biotype:protein_coding]	FBL

Table S2. The potential targets of EI.

Compounds	Targets	Source	Symbol
beta-elemene	Alpha-1A adrenergic receptor	TCMSP	ADRA1A
beta-elemene	Apoptosis regulator Bcl-2	TCMSP	BCL2
beta-elemene	G2/mitotic-specific cyclin-B1	TCMSP	CCNB1
beta-elemene	Cell division control protein 2 homolog	TCMSP	CDK1
beta-elemene	Cyclin-dependent kinase inhibitor 1	TCMSP	CDKN1B
beta-elemene	Muscarinic acetylcholine receptor M1	TCMSP	CHRM1
beta-elemene	Muscarinic acetylcholine receptor M2	TCMSP	CHRM2
beta-elemene	Muscarinic acetylcholine receptor M3	TCMSP	CHRM3
beta-elemene	Neuronal acetylcholine receptor protein, alpha-7 chain	TCMSP	CHRNA7
beta-elemene	Gamma-aminobutyric acid receptor subunit alpha-1	TCMSP	GABRA1
beta-elemene	Gamma-aminobutyric-acid receptor alpha-2 subunit	TCMSP	GABRA2
beta-elemene	Gamma-aminobutyric-acid receptor alpha-3 subunit	TCMSP	GABRA3
beta-elemene	Gamma-aminobutyric-acid receptor alpha-5 subunit	TCMSP	GABRA5
beta-elemene	Gamma-aminobutyric-acid receptor subunit alpha-6	TCMSP	GABRA6
beta-elemene	Nuclear receptor coactivator 2	TCMSP	NCOA2
beta-elemene	Prostaglandin G/H synthase 1	TCMSP	PTGS1
beta-elemene	Prostaglandin G/H synthase 2	TCMSP	PTGS2
beta-elemene	Retinoblastoma-associated protein	TCMSP	RBL1
beta-elemene	Transforming protein RhoA	TCMSP	RHOA
beta-elemene	Protein CBFA2T1	TCMSP	RUNX1T1
beta-elemene	Retinoic acid receptor RXR-alpha	TCMSP	RXRA
beta-elemene	Sodium-dependent noradrenaline transporter	TCMSP	SLC6A2
beta-elemene	Telomerase protein component 1	TCMSP	TEP1
beta-elemene	Eukaryotic translation initiation factor 6	TCMSP	TIF6
beta-elemene	Cellular tumor antigen p53	TCMSP	TP53
beta-elemene	BAX	CTD	BAX
beta-elemene	BCL2	CTD	BCL2
beta-elemene	CASP3	CTD	CASP3
beta-elemene	CASP9	CTD	CASP9
beta-elemene	CAT	CTD	CAT
beta-elemene	CCND1	CTD	CCND1
beta-elemene	IL6	CTD	IL6
beta-elemene	NOS2	CTD	NOS2

Compounds	Targets	Source	Symbol
beta-elemene	PCNA	CTD	PCNA
beta-elemene	PTGS2	CTD	PTGS2
beta-elemene	TNF	CTD	TNF
delta-elemene	Alpha-1B adrenergic receptor	TCMSP	ADRA1B
delta-elemene	Alpha-1A adrenergic receptor	TCMSP	ADRA2A
delta-elemene	Caspase-3	TCMSP	CASP3
delta-elemene	Muscarinic acetylcholine receptor M1	TCMSP	CHRM1
delta-elemene	Muscarinic acetylcholine receptor M2	TCMSP	CHRM2
delta-elemene	Muscarinic acetylcholine receptor M3	TCMSP	CHRM3
delta-elemene	Neuronal acetylcholine receptor protein, alpha-7 chain	TCMSP	CHRNA7
delta-elemene	Gamma-aminobutyric acid receptor subunit alpha-1	TCMSP	GABRA1
delta-elemene	Gamma-aminobutyric-acid receptor alpha-2 subunit	TCMSP	GABRA2
delta-elemene	Gamma-aminobutyric-acid receptor alpha-3 subunit	TCMSP	GABRA3
delta-elemene	Gamma-aminobutyric-acid receptor subunit alpha-6	TCMSP	GABRA6
delta-elemene	Nuclear receptor coactivator 2	TCMSP	NCOA2
delta-elemene	Prostaglandin G/H synthase 1	TCMSP	PTGS1
delta-elemene	Prostaglandin G/H synthase 2	TCMSP	PTGS2
delta-elemene	Retinoic acid receptor RXR-alpha	TCMSP	RXRA
delta-elemene	Sodium-dependent noradrenaline transporter	TCMSP	SLC6A2
gamma-elemene	Gamma-aminobutyric acid receptor subunit alpha-1	TCMSP	GABRA1
gamma-elemene	Gamma-aminobutyric-acid receptor alpha-2 subunit	TCMSP	GABRA2
gamma-elemene	Gamma-aminobutyric-acid receptor alpha-3 subunit	TCMSP	GABRA3
gamma-elemene	Gamma-aminobutyric-acid receptor subunit alpha-6	TCMSP	GABRA6
gamma-elemene	Nuclear receptor coactivator 2	TCMSP	NCOA2
gamma-elemene	Prostaglandin G/H synthase 2	TCMSP	PTGS2
gamma-elemene	Retinoic acid receptor RXR-alpha	TCMSP	RXRA
beta-elemene		STP	CXCR3
beta-elemene		STP	HTR2A
beta-elemene		STP	MAOB
beta-elemene		STP	CYP1A2
beta-elemene		STP	MGLL
beta-elemene		STP	HSD11B1
beta-elemene		STP	BCHE
beta-elemene		STP	MAOA

Compounds	Targets	Source	Symbol
beta-elemene		STP	ACHE
beta-elemene		STP	SLC6A2
beta-elemene		STP	HTR2C
beta-elemene		STP	PTGS2
beta-elemene		STP	UGT2B7
beta-elemene		STP	ESR2
beta-elemene		STP	FADS1
beta-elemene		STP	PPARA
beta-elemene		STP	CNR2
beta-elemene		STP	SHBG
beta-elemene		STP	NR1H3
beta-elemene		STP	CYP19A1
beta-elemene		STP	PTPN1
beta-elemene		STP	NR1I3
beta-elemene		STP	PTGS1
delta-elemene		STP	PPARA
delta-elemene		STP	CNR2
delta-elemene		STP	NR1H3
delta-elemene		STP	CXCR3
delta-elemene		STP	HTR2A
delta-elemene		STP	ADORA1
delta-elemene		STP	ADORA2A
delta-elemene		STP	ADORA3
delta-elemene		STP	AR
delta-elemene		STP	ESR1
delta-elemene		STP	ALOX5
delta-elemene		STP	PTPN2
delta-elemene		STP	CYP19A1
delta-elemene		STP	TRPV1
delta-elemene		STP	MAOB
delta-elemene		STP	GLI2
delta-elemene		STP	GLI1
delta-elemene		STP	FAAH
delta-elemene		STP	CHRM2

Compounds	Targets	Source	Symbol
delta-elemene		STP	ACHE
delta-elemene		STP	SLC6A2
delta-elemene		STP	SLC6A4
delta-elemene		STP	CYP2C19
delta-elemene		STP	CYP1A2
delta-elemene		STP	SQLE
delta-elemene		STP	BCHE
delta-elemene		STP	PTPN1
delta-elemene		STP	PIN1
delta-elemene		STP	NR1I3
delta-elemene		STP	CNR1
delta-elemene		STP	FABP4
delta-elemene		STP	PTGS1
delta-elemene		STP	PPARG
delta-elemene		STP	FABP3
delta-elemene		STP	PPARD
delta-elemene		STP	FFAR1
delta-elemene		STP	ESR2
delta-elemene		STP	TOP1
delta-elemene		STP	PLA2G4A
delta-elemene		STP	SHBG
delta-elemene		STP	RORC
delta-elemene		STP	SREBF2
delta-elemene		STP	NPC1L1
delta-elemene		STP	CTSD
delta-elemene		STP	CYP17A1
delta-elemene		STP	HMGCR
delta-elemene		STP	CYP51A1
delta-elemene		STP	POLB
delta-elemene		STP	SRD5A1
delta-elemene		STP	SRD5A2
delta-elemene		STP	NR3C2
delta-elemene		STP	NR3C1
delta-elemene		STP	PGR

Compounds	Targets	Source	Symbol
delta-elemene		STP	SERPINA6
delta-elemene		STP	SIGMAR1
delta-elemene		STP	NR1I2
delta-elemene		STP	PLA2G4B
delta-elemene		STP	HSD17B3
delta-elemene		STP	PLA2G4C
delta-elemene		STP	FABP1
delta-elemene		STP	KMT5A
delta-elemene		STP	RARG
delta-elemene		STP	RARB
delta-elemene		STP	RARA
delta-elemene		STP	RBP4
delta-elemene		STP	PTPN6
delta-elemene		STP	PLK1
delta-elemene		STP	LSS
delta-elemene		STP	TERT
delta-elemene		STP	FABP5
delta-elemene		STP	RXRA
delta-elemene		STP	UGT2B7
delta-elemene		STP	RXRG
delta-elemene		STP	CES2
delta-elemene		STP	PREP
delta-elemene		STP	PTGES
delta-elemene		STP	CES1
delta-elemene		STP	SCD
delta-elemene		STP	MGLL
delta-elemene		STP	NOS2
delta-elemene		STP	RORA
gamma-elemene		STP	PPARA
gamma-elemene		STP	CNR2
gamma-elemene		STP	CXCR3
gamma-elemene		STP	NR1H3
gamma-elemene		STP	HTR2A
gamma-elemene		STP	MAOB

Compounds	Targets	Source	Symbol
gamma-elemene		STP	UGT2B7
gamma-elemene		STP	MGLL
gamma-elemene		STP	CYP19A1
gamma-elemene		STP	NR1I3
gamma-elemene		STP	FAAH
gamma-elemene		STP	TRPV1
gamma-elemene		STP	AR
gamma-elemene		STP	ESR1
gamma-elemene		STP	CHRM2
gamma-elemene		STP	ACHE
gamma-elemene		STP	SLC6A2
gamma-elemene		STP	SLC6A4
gamma-elemene		STP	CYP2C19
gamma-elemene		STP	SQLE
gamma-elemene		STP	BCHE
gamma-elemene		STP	PTPN1
gamma-elemene		STP	CYP1A2
gamma-elemene		STP	PIN1
gamma-elemene		STP	PTPN2
gamma-elemene		STP	TRPA1
gamma-elemene		STP	PTGS1
gamma-elemene		STP	TOP1
gamma-elemene		STP	ADORA1
gamma-elemene		STP	ADORA2A
gamma-elemene		STP	ADORA3
gamma-elemene		STP	SHBG
gamma-elemene		STP	RORC
gamma-elemene		STP	SREBF2
gamma-elemene		STP	NPC1L1
gamma-elemene		STP	CTSD
gamma-elemene		STP	CYP17A1
gamma-elemene		STP	CDC25A
gamma-elemene		STP	HMGCR
gamma-elemene		STP	CDC25B

Compounds	Targets	Source	Symbol
gamma-elemene		STP	CYP51A1
gamma-elemene		STP	SRD5A1
gamma-elemene		STP	SRD5A2
gamma-elemene		STP	NR3C2
gamma-elemene		STP	NR3C1
gamma-elemene		STP	PGR
gamma-elemene		STP	SERPINA6
gamma-elemene		STP	SIGMAR1
gamma-elemene		STP	NR1I2
gamma-elemene		STP	HSD17B3
gamma-elemene		STP	FABP1
gamma-elemene		STP	VDR
gamma-elemene		STP	ALOX5
gamma-elemene		STP	ATP12A
gamma-elemene		STP	PTPN6
gamma-elemene		STP	HSD11B1
gamma-elemene		STP	PTGS2
gamma-elemene		STP	CCR5
gamma-elemene		STP	FABP4
gamma-elemene		STP	PPARG
gamma-elemene		STP	TERT
gamma-elemene		STP	FABP3
gamma-elemene		STP	FABP5
gamma-elemene		STP	PPARD
gamma-elemene		STP	CNR1
gamma-elemene		STP	GLI2
gamma-elemene		STP	GLI1
gamma-elemene		STP	ESR2
